# Supplementary material for: Positive feedbacks and alternative stable states in forest leaf types
Source: Nat Commun. 2024 May 31;15:4658. doi: 10.1038/s41467-024-48676-5 (PMC11143268; doi:10.1038/s41467-024-48676-5)
Supplement: Supplementary file 1 — Supplementary Information [file 41467_2024_48676_MOESM1_ESM.pdf]

Supporting Information for

Positive feedbacks and alternative stable states in forest leaf types

Email: yibiao.zou@usys.ethz.ch

Supplementary Note 1: Bimodality testing with control for overdispersion.

Overdispersion means that the distribution of individuals in plots is aggregated, leading to more heavily populated but also more empty plots, and thus a higher variance than expected by a Poisson distribution[1]. A negative binomial distribution allows to account for overdispersion by including an additional overdispersion parameter. Reasons for overdispersion include dispersal limitation or mother-offspring relationships. Because count data also often shows more or fewer zeros than expected from the underlying distribution, zero-adjusted models are needed for accurate predictions[2].

To control for overdispersion, we again fit GAMLSS models to the plot-level absolute abundance of evergreen and deciduous trees, respectively, but with a zero-adjusted negative binomial error distribution (Fig. S3). Similarly, all parameters of the distribution (mean parameter with log link, dispersion parameter with log link, zero probability parameter with logit link) were modelled as a function of the ten environmental principal components. The rest of the setting are the same as in the main text (Materials and Methods: Bimodality testing).

Supplementary Note 2: Demographic models: extended analysis.

Species-level demographic models.

We fit demographic models as described in the main text (Methods: Demographic models and simulations) but at the species level for the 10 most abundant evergreen and 10 most abundant deciduous tree species in terms of the number of individual stems within the FIA dataset. Model structure and covariates were identical, except for recruitment, where con-phenological basal area was replaced with the conspecific basal area within a plot.

Ecoregion-level demographic models.

The FIA data contains many ecological subregions with distinct tree species composition and potentially unique interactions between evergreen and deciduous functional groups. To understand if our findings across the United States are consistent within ecological subregions, we refit growth, recruitment and mortality probability models as described in the main text to five regional subsets of the dataset in the eastern US. We selected regional subsets using EPA level 2 ecoregions[3], with each subregion containing at least 1,000 unique forest inventory plots.

European demographic models.

Tree species diversity (and mixing) differs between North America and Europe. To test our observations and predictions arising from the North American data on an entirely independent dataset, we additionally fit demographic GAMs to the European data, modelling recruitment, growth and survival of evergreen and deciduous trees.

Supplementary Note 3: Optimization and evaluation of spatial random forest models.

Grid-size tuning.

To determine the optimal grid size of the spatial "fishing net", we trained a series of random forest models using grid sizes from 0.01 to 2 degree. The results showed that a grid size of 1/6 degree (~20 km) results in the most accurate random forest model, with the highest prediction R^2 and lowest RMSE (Fig. S15).

Hyperparameter tuning.

To train global random forest models of bimodality in forest leaf phenology, we first ran a hyperparameter-tuning procedure, exploring the results of a set of models with different hyperparameters. We varied the number of variables sampled at each split (1, 2, 4, 5, 8, 10, 15 and 20) and the minimum sample size at the end of the nodes (1, 2, 5, 10 and 20). We used coefficients of determination (R^2) and the root mean square error (RMSE) based on 10-fold cross-validation as the model evaluation metric. The best-tuned model was used to generate the map shown in Fig. 4.

Bootstrapping analysis.

To evaluate model uncertainty from the second partition, we implemented a stratified bootstrapping procedure[4,5]. With 100 bootstrap iterations, we sampled the training data with replacement, using biome as the stratification criterion to proportionally reflect the major bioclimatic zones in each of the 100 bootstrap samples. The size of each sampling dataset corresponded to the size of the original dataset. As a measure of prediction uncertainty, we calculated the standard deviation of predictions for each pixel across the 100 bootstrap layers.

Interpolation vs. Extrapolation analysis.

To evaluate how well our training dataset represents the full multivariate environmental covariate space, we performed a principal-component-analysis-based approach following van den Hoogen et al. (2021). For the PCA-based approach, we projected the covariate composite into the same space using the standardized values and eigenvectors from the principal component analysis of the training data. We created the convex hulls for each of the bivariate combinations from the top principal components and classified whether each pixel falls in or outside each of these convex hulls (Fig. S9B). We used 22 principal components with 68 combinations for all covariates for the sampling dataset. To provide validation for this method, we applied two other methods for interpolation vs. extrapolation analysis. In the first method, we created convex hulls using only the leading (two, three or four) principal components (we decided on a maximum number of four principal components due to computational limitation). We then checked whether each pixel fell in or outside each of these high dimensional convex hulls (Fig. S31). In the second method, for each pixel, we computed the percentage of all 62 covariates with values falling into the range of our training data (Fig. S9A). All methods agree high extrapolation risks in tropical regions.

Spatial leave-one-out cross validation.

To account for the potential effect of spatial autocorrelation in model residuals from the random forest model (based on the spatial clustering procedure), we ran spatially buffered leave-one-out cross validation (SLOO-CV) for a series of buffer radii from 0 km to 1,000 km. In SLOO-CV, each observation is predicted based on a model that includes all data outside the respective buffer radius, resulting in ~15,000 separate random forest models for each buffer radius. Model performance was evaluated based on R^2 and RMSE. We also plotted semi-variograms for model residuals of both random cross validation and SLOO-CV (Fig. S12). To provide a reference for the full model with 62 covariates, we trained a purely spatial model (null model) using only latitude and longitude to predict BI. We then applied the same SLOO-CV procedure on the null model.

Supplementary Note 4: Determinant analysis: extended analysis.

To further confirm the results as in Fig. 5, we implemented a similar procedure but using environmental principal components as predictors. We implemented principal component analysis on three groups of abiotic environmental covariates, namely 30 climatic, 10 soil, 12 topographic variables, and selected the first 3 principal components for each group, resulting in a total of 9 predictors ($PC1_{climate}$, $PC2_{climate}$, $PC3_{climate}$, $PC1_{soil}$, etc.). The first random forest model fit the forest bimodality index as a function of the 9 principal components at the global scale. The second set of models fit plot-level relative evergreen abundance as a function of the 9 principal components for all plots within forest grids with bimodal distribution (namely BI in -0.22 ~ 0.22), evergreen-dominated distribution (BI > 0.22) or deciduous-dominated distribution (BI < -0.22). We then calculated the permutation importance of each variable used in the random forest models (Fig. S11).

Supplementary Note 5: Testing the effects of forest successional status and monoculture.

Several forest plots incorporated into our study could potentially be in a successional phase. Consequently, their present phenological type composition may be unstable and susceptible to eventual replacement by other phenological types. This factor introduces potential bias in our analysis results as these transitory forest plots do not represent the ultimate stable states. In addition, during data cleaning for our primary analysis, we excluded monoculture plots from the GFBi and FunDivEUROPE datasets to account for human management effects. Nevertheless, this approach might inadvertently exclude some valid plots as certain natural forests may also exhibit monoculture characteristics.

To address these concerns, we conducted an additional analysis similar to the methods illustrated in Fig. 1 and Fig. 2, employing late successional phase forest data and retaining monoculture plots. Existing research suggests that the Diameter at Breast Height (DBH) distribution within a plot can serve as an indicator of both the successional stage and the age of the forest[6,7]. Given the unavailability of forest age data for all our plots, we utilized the plot-mean DBH as a proxy for the

successional stage and the age of the forest. We initially computed the plot-mean DBH for all GFBi plots, from which we derived the 0.75 quantile of plot-mean DBH, approximately 25 cm. We then filtered the GFBi, FIA, and FunDivEUROPE datasets to retain only those plots where the plot-mean DBH exceeded 25 cm, a criterion we used to define late-successional (and thus, stable) plots. All monoculture plots within these three datasets were retained for analysis.

Subsequently, we replicated the bimodality testing across all three datasets, and the demographic analysis was conducted using the FIA dataset. The outcomes (Fig. S20) demonstrated overall consistency with our primary analysis (Fig. 1-2).

Supplementary Note 6: Recruitment analysis with correction for seed source.

The pattern observed in Fig. 2C, specifically the higher recruitment rates amongst trees surrounded by their own phenological types, may be largely attributable to the availability of seed sources. For instance, a dominant species within a plot is likely to establish a substantial seed source and, as a consequence, have a higher probability of successful recruitment than other species. Given that a dominant species can either be evergreen or deciduous, this recruitment advantage might contribute to the observed trend of higher recruitment rates for evergreen trees in evergreen-dominated forests, and similarly for deciduous trees in deciduous forests. While the seed source indeed contributes to the positive feedback within phenological types, we sought to evaluate if this recruitment feedback persists even when the seed source advantage of the dominant species is accounted for.

In an attempt to control for the impact of seed source, we excluded data of the dominant species, identified as those with the most substantial number of individuals in each FIA plot. Furthermore, we retained only those forest plots where the relative evergreen abundance ranged between 0.1-0.9, ensuring that both evergreen and deciduous trees established local seed sources within each plot. Following this, we replicated the recruitment modeling illustrated in Materials and Methods (Demographic models and simulations: Modelling tree recruitment). The results (Fig. S21) exhibited patterns consistent with our primary findings (Fig. 2C).

We must acknowledge that it is challenging to fully disentangle seed availability from other feedback mechanisms, such as plant-soil interactions. While seed availability does contribute to the feedback loop, where evergreens foster more evergreens, our study reveals a bimodality consistent with bifurcation (as depicted in Figures 1-3) that cannot be explained solely by climate or soil characteristics. Instead, this bimodality is dependent on initial conditions, exhibiting hysteresis (Fig. 3E-F). The presence of a strong hysteresis effect implies that a simple recruitment mechanism based solely on seed availability may not be sufficient to generate such a potent feedback loop. This finding and our extended analysis here (Fig. S21) suggest that other factors, beyond seed availability, are likely involved in shaping and sustaining the observed bimodality and hysteresis.

Supplementary Note 7: Validating random forest analysis using point-level soil data.

The Soil Grids dataset, in order to create global soil feature layers, employs machine learning models guided by comprehensive, spatially-explicit data on various climatic factors. This method of soil information interpolation using climatic data potentially leads to an overemphasis on the covariance between climate and soil layers, while diminishing the microscale heterogeneity of soil features. In order to examine whether this potential limitation impacts our findings, we incorporated point-level soil measurements from the World Soil Information Service (WOSIS) dataset[8]. This dataset included soil clay content, silt content, pH, sand content, nitrogen density, and coarse volumetric fraction. In order to spatially align this data with the exhaustive GFBi dataset, which comprises over 1.1 million plots globally, we chose the closest soil observation within a radius of either 250 m or 1000 m from each forest plot's center. This yielded a spatial correspondence between soil measurements and forest plots in 182 instances for the 250 m radius and in 2,346 instances for the 1000 m radius (Fig. S22A).

For the purpose of validating the consistency between results obtained from global soil layers and point-level soil observations, we executed a supplementary random forest analysis using the alignment with both the 250 m radius and the 1000 m radius. The outcomes of these analyses confirm that: i) model predictions remain substantially unchanged when point observations substitute global soil layers (with a 97% similarity in predictions of 10-fold cross-validation, as depicted in Fig. S23B & S24B); ii) a high degree of agreement exists between global layers and point observations for the majority of soil variables (Fig. S22); and iii) the relative importance of soil features in phenological type variation amongst differing cluster types is generally maintained when point observations are used in lieu of soil layers as predictors (Fig. S23A, C & S24A, C). These analyses further accentuate the critical influence of soil features in shaping global forest phenological variations.

Supplementary Note 8: Spatial and environmental analysis for forest plots in bimodal clusters.
As outlined in the main body of the text, we segmented the GFBi dataset using a 10 arc-min (20km) grid-based 'fishing net' approach to establish spatial clusters (totaling 14,931), and computed a cluster-level Bimodality Index (BI) based on the forest plot information within each cluster. Subsequently, we classified these clusters into bimodal clusters (BI = -0.22~0.22), evergreen-dominated clusters (BI > 0.22), and deciduous-dominated clusters (BI < -0.22). The existence of bimodal clusters (comprising 12% of all clusters) supports the hypothesis that stable evergreen and deciduous forests can coexist in close proximity within each 20km x 20km grid cell, forming distinct patches via positive feedback mechanisms, rather than environmental filtering. This assertion can be further substantiated by an explicit exploration of the spatial distribution and environmental variation of forest plots within each bimodal cluster.

Comparison between spatial and environmental grouping.
To critically assess the consistency of the pattern, i.e., forests forming spatial patches of similar phenological type in environmentally alike regions across all bimodal clusters, we conducted an exhaustive multi-step analysis. This analysis aimed to: 1) confirm whether evergreen and deciduous trees segregate into distinct spatial patches within bimodal clusters and 2) investigate if environmental factors fully account for this patch formation.

To attain these objectives, we first classified each forest plot within every bimodal cluster into one of three categories—evergreen, mixed, or deciduous—based on plot-level relative Evergreen Vegetation Index (relEV). Subsequently, we compared these predefined categories with five different groupings, which were established based on plot spatial coordinates, environmental conditions (inclusive of both climate and soil), randomly shuffled environmental conditions (serving as a null group), climatic conditions, and soil information. The concurrence between predefined categories and spatial location groupings would indicate distinct spatial patch formations of evergreen and deciduous forests. Similarly, strong alignment between predefined categories and environmental groupings would suggest environmental factors influencing patchy distributions.

The specific procedural steps are as follows:
Step 1: Classify each forest plot within a bimodal cluster into one of three categories based on plot-level relEV: evergreen (relEV > 0.66), mixed (relEV = 0.33-0.66), and deciduous (relEV < 0.33).
Step 2: Implement the K-means method[9] to categorize forest plots into three spatial classes within each bimodal cluster, based on their latitude and longitude coordinates. This method groups geographically proximal plots into one class.
Step 3: Execute a Principal Component Analysis (PCA) on 62 environmental covariates for each cluster (refer to Table S2 for details), followed by K-means classification into three environmental groups based on the leading five environmental Principal Components (PCs). This method groups environmentally similar plots into one class.
Step 4: After the PCA in Step 3, randomize the order of the top ten environmental PCs across all plots and categorize the plots into three environmental classes using the K-means method, but this time based on randomized PCs.
Step 5: Conduct a PCA on 26 climatic covariates for each cluster, followed by K-means categorization into three climatic groups based on the leading five climatic PCs. This method groups climatically similar plots into one class.
Step 6: Perform a PCA on 9 soil covariates for each cluster, followed by K-means categorization into three soil groups based on the leading five soil PCs. This method groups plots with similar soil conditions into one class.
Step 7: To compare the five types of groupings (spatial, environmental, random, climate, and soil) with our predefined categories based on forest phenological composition, we employed the Adjusted Rand Index (ARI), a standard metric for comparing two partitions[10]. ARI ranges from 0 to 1, with higher values indicating better alignment between two partitions.

We repeated these steps for each bimodal cluster, yielding five ARIs per cluster. The ARI distributions were then visualized for further interpretation. Our results showed that spatial grouping yielded the highest ARI, significantly surpassing values from the other four methods, followed by soil grouping (Fig. S26). This finding strongly suggests that different forest phenological types form distinct spatial patches. The comparatively higher ARI for environmental grouping than the random group implies that environmental heterogeneity partially impacts the distribution of phenological types but does not entirely account for patch formation, as the overall match remains poor (Fig. S26). Moreover, the elevated ARI for soil grouping over climatic grouping suggests that soil variance better explain the patch formation than climate.

Visual illustration with examples.
To offer a clear illustration of the spatial arrangement and environmental variation of plots in bimodal clusters, we randomly selected three such clusters as examples (Fig. S25A, two located in the US and one in Europe). For each cluster, we depicted the coordinates of forest plots within that cluster (Fig. S25B-D), and presented the histogram of plot-level relative evergreen abundance, showcasing a bimodal distribution in all three clusters (Fig. S25H-J). Additionally, we conducted a principal component analysis (PCA) on 62 environmental covariates for each cluster (Refer to Table. S2 for a detailed description), and plotted the locations of forest plots on the environmental space delineated by the leading two principal

components (Fig. S25E-G). The results indicated that evergreen forests are spatially closer to other evergreens, and deciduous forests are in closer proximity to other deciduous forests (Fig. S25B-D). However, no clear clustering of evergreen or deciduous forests was evident in the PCA space, implying these forests inhabit similar environmental conditions (Fig. S25E-G).

Comparison of environmental variance among three types of clusters.

Should climate variance primarily drive bimodality, we would expect to observe heightened climatic variance in bimodal clusters compared to deciduous-dominated or evergreen-dominated clusters. Conversely, if plant-soil interactions mainly influence bimodal patterns, higher soil variance would be expected in bimodal clusters relative to the other two types of clusters. We conducted a comparative analysis to test these hypotheses, evaluating the variance of both climatic and soil covariates, such as temperature, precipitation, and soil pH, among all plots within each cluster of the three cluster types. Our results indicate a lower climatic variance within bimodal clusters compared to both deciduous-dominated and evergreen-dominated clusters. Contrarily, we observed higher soil variance within bimodal clusters relative to the other cluster types (see Fig. S27). This evidence suggests that bimodal patterns are not primarily driven by increased climatic variance; rather, they are more likely a result of plant-soil interactions that produce heightened soil variance within bimodal clusters.

In summary, these detailed analyses support the assertion that forests form spatial patches of their own phenological type in environmentally similar areas within bimodal clusters. This observed pattern is likely the outcome of positive feedback mechanisms (e.g., plant-soil interactions), given the inadequacy of environmental filtering to explain such formations. Further, this interpretation is bolstered by evidence of demographic positive feedbacks presented in the main text (Fig. 2).

Supplementary Note 9: Comparison between random forest model predictions and a remote sensing product.

To compare our model predictions with 10 m resolution remote sensing data for Europe (https://land.copernicus.eu/en/products/high-resolution-layer-dominant-leaf-type/dominant-leaf-type-2018#download) for validation, we implemented the following procedure. Given this dataset only includes information of coniferous and broadleaf forests, we first treated coniferous as evergreen, and broadleaf as deciduous. We also approximated each pixel as one canopy. We then computed the percentage of coniferous pixels across all forest pixels within each 100m*100m grid. Next, we computed BI based all 100m resolution grid within each 20km*20km grid. The scatter plot between remote sensing observations and predictions from spatial random forest model shows an R^2 of 0.35, which is quite good (Fig. S32A). And the observations and predictions match well in bimodal regions (Bis in -0.22 - 0.22) and evergreen-dominated regions (BIs > 0.22). We can also see that the model predicts more evergreen-dominated plots when remote-sensing showing they are “deciduous-dominated plots”. This discrepancy mainly results from our treatment of approximating broadleaf as deciduous, therefore ignoring the presence of broadleaf-evergreen forests. To account for this limitation, we use the 300m resolution forest type map (https://cds.climate.copernicus.eu/cdsapp#!/dataset/10.24381/cds.006f2c9a?tab=overview) to filter the 10m resolution dataset to only retain needleleaf-evergreen, broadleaf-deciduous and mixed forests. We then apply the same comparison procedure as above. The result showed way better agreement between data and model (R^2=0.44, Fig. S32B).

Supplementary Note 10: Testing bimodality when treating leaf shedding as a continuous trait.

We recognize the potential limitations of categorizing species strictly as either evergreen or deciduous, a dichotomy that simplifies the complex spectrum of leaf shedding behaviors across different species. For example, a hypothetical tree that sheds leaves for one month per year is currently treated the same as a tree that sheds them for 11 months.

To test this, we examined whether this bimodality trend is apparent if we treat deciduous as a continuous or categorical trait. In this case we found that, whether deciduousness is treated as a category, or a continuous trait does not affect the bimodal distribution between evergreen and deciduous species. As such, it has no bearing on the overall conclusions. To demonstrate that the actual underlying distribution of leaf shedding, or deciduousness, is bimodal, we now used the most comprehensive species-level dataset on growing season length from Zohner et al. (2017), which includes data for more than 400 species from North America, Europe and Asia[11]. Fig. S33 shows that across all continents, there is a clear bimodal distribution, which was also statistically confirmed by the significant P values of a Hartigan’s dip test.

Moreover, it is important to clarify that our study's focus is on regions with pronounced seasonal variations (latitudes greater than 15° N), where the distinction between deciduous and evergreen species is not only clear but also rooted in their genetic makeup. For example, deciduous trees, such as beeches, invariably lose their leaves in response to winter conditions, while evergreens, like spruces, retain their foliage throughout the year. This genetically determined behavior results in

predictable patterns of leaf phenology that are consistent across individuals of the same species, despite spatial variations in the length of the leafless season.

Our study's scope is specifically tailored to understand these genetically fixed phenological patterns within a clear seasonal framework. Including additional variables, such as the precise length of the growing season for each species, while potentially enriching in a different context, would not alter the classification of a species as deciduous or evergreen within the studied latitudes. This is because, in the regions we are examining, an evergreen tree's inherent genetic traits ensure its evergreen status, and similarly, a deciduous tree remains deciduous regardless of slight variations in the growing season's length.

Our decision to adhere to this dichotomous classification system is driven by the genetic demarcation between deciduous and evergreen species in these latitudes, coupled with the practical limitations of data availability. Extending our analysis to account for the nuanced length of the growing season across the vast geographic and climatic spectrum we are studying would necessitate a level of detailed phenological data that is currently beyond our reach. Adding information on the precise growing season length of each individual in each plot is simply not possible. Species-level information would not add much to this question, as there is too much geographic variation within species, which is often higher than the variation across species.

Supplementary Note 11: Testing the correlation between leaf phenology strategies, mycorrhizal types and leaf forms.
In recent years, a growing body of research supports the important role of soil communities in shaping such plant-soil feedbacks. For example, Averill et al. 2022 discovered similar feedback mechanisms that lead to the emergence of bimodality in forest mycorrhizal types, suggesting that alternative stable states drive forest composition by affecting the distribution of arbuscular and ectomycorrhizal trees[12]. An important question is whether these feedback mechanisms are linked to interactively shape the spatial variation of forest trees. However, we found no significant association between leaf phenology type and mycorrhizal type based on US FIA data set (Fisher's Exact Test $p = 0.99$, Table. S1). Among all ectomycorrhizal-associated species in the FIA dataset, 53% are evergreen trees, and 43% are deciduous (Table. S1). Within arbuscular species, 69% are evergreen, and 31% are deciduous. In addition, geographic variation in the bimodality of mycorrhizal types explains only 10% of the distribution of leaf phenology strategies, based on the GFBi data (Fig. S13A). These results support the independence of alternative stable states, suggesting that independent feedback mechanisms drive the observed bimodality in forest mycorrhizal and phenological strategies. Yet, whether they can interact in certain areas seems a promising research question given that both are strongly linked to soil pH and nutrients. In contrast, geographic variation in the bimodality of leaf form (needleleaf versus broadleaf) can explain 72% of the distribution of leaf phenology strategies based on the GFBi data (Fig. S13B), and the bimodality in leaf phenology strategies thus mainly stems from the bimodal patterns of needleleaf evergreen vs. broadleaf deciduous forests.

Supplementary Note 12: Testing for residual spatial autocorrelation of tree recruitment.
To test for residual spatial autocorrelation of tree recruitment, we first fit a recruitment model with only an intercept (raw model), and plotted semivariance of residuals against distance for both deciduous and evergreen trees. We then fit a recruitment model using GAM and plotted the semivariogram for both phenological strategies. The results suggest that dispersal limitation affects tree recruitment, as the semivariance of residuals increases with distance (Fig. S2). The final statistical models were able to capture and account for this autocorrelation and therefore could separate local positive feedbacks from the potential influence of dispersal limitation.

Supplementary Figures

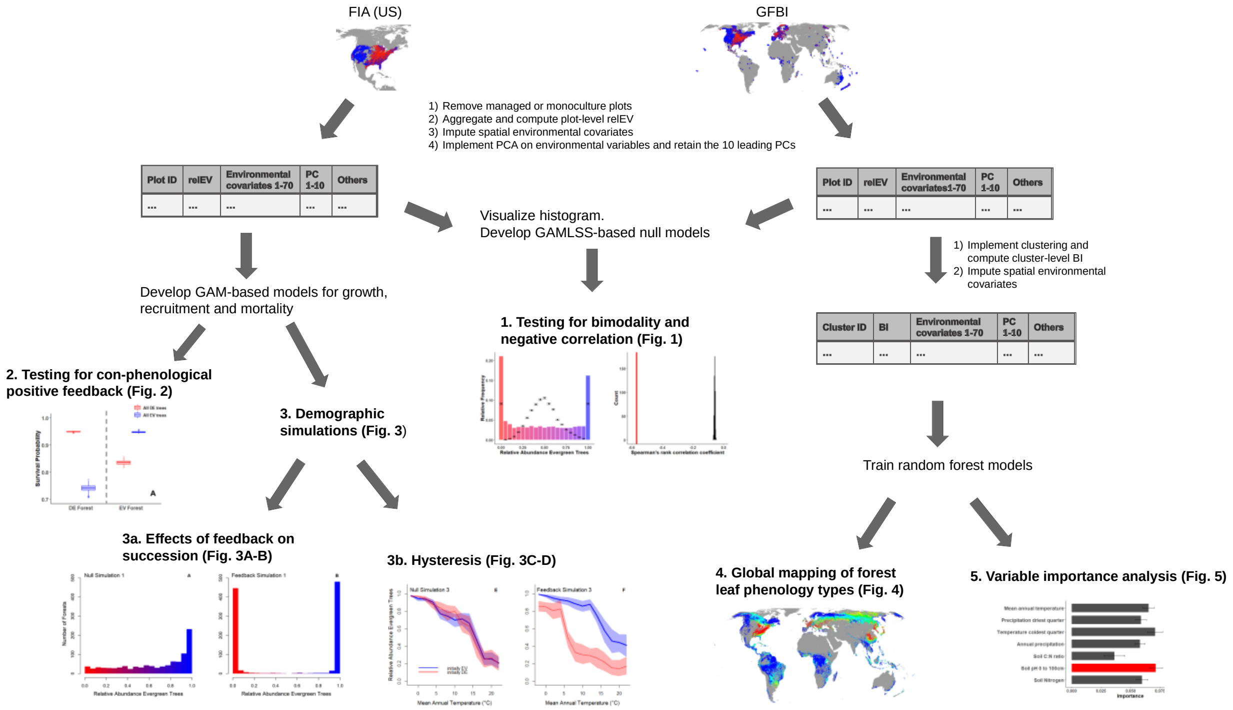

Fig. S1 | Overview of the analyses conducted in this study. We used forest inventory plot data with detailed forest composition information: the GFBi dataset for global scale analysis, FIA data for analysis of mainland US, and FunDivEUROPE data for analysis of Europe. The FIA and FunDivEUROPE have repeated measurements, which we used for demographic analysis. For all three datasets, we removed managed/monoculture plots to control for human impacts. We then calculated plot-level relative evergreen abundance (relEV) and extracted spatial environmental covariates for each forest plot. In the next step, we conducted a PCA for those spatial environmental covariates and retained the ten leading PCs. With these preprocessed data, we tested the four criteria of alternative stable states described in the introduction. In addition, we used random forest models to map the bimodality in forest leaf phenology types across the global forest extent. We then calculated variable importance to quantify the key factors that drive these biogeographic patterns. The first three analyses can provide evidence for the existence of alternative stable states. The fourth analysis quantifies the global extent of alternative stable states and the key drivers.

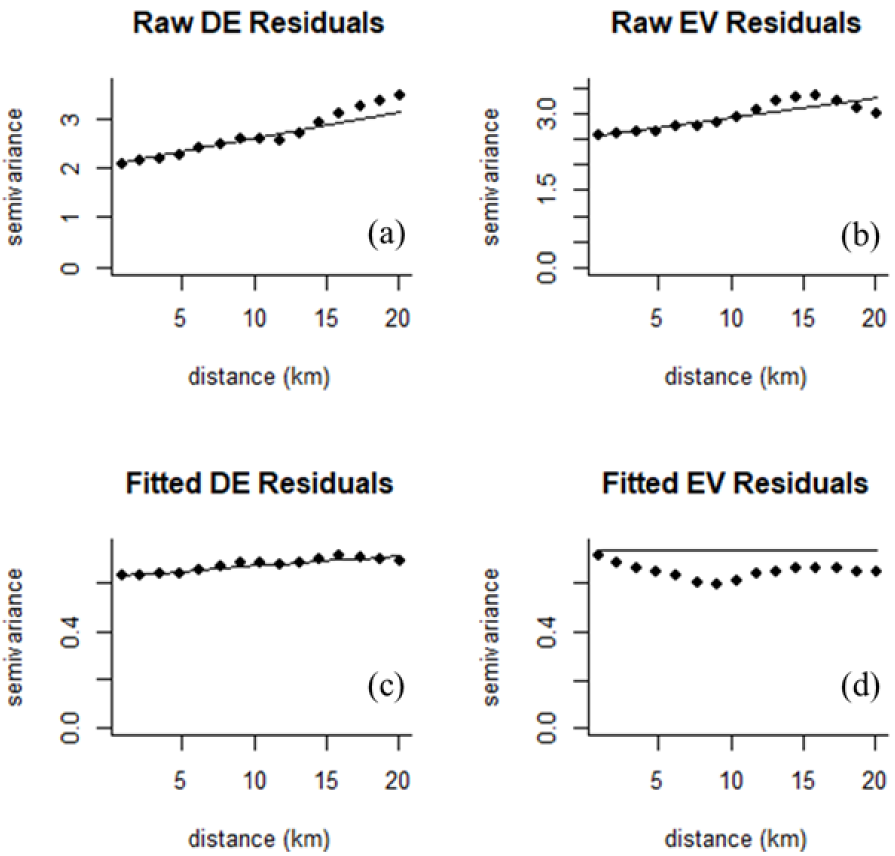

Fig. S2 | Spatial autocorrelation of model residuals. Residual spatial autocorrelation, visualized as semi-variance as a function of spatial distance in (a) residuals of deciduous (DE) tree recruitment modelled with only an intercept, (b) residuals of evergreen (EV) tree recruitment modelled with only an intercept, (c) residuals of deciduous tree recruitment in the full GAM model, and (d) residuals of evergreen tree recruitment in the full GAM model.

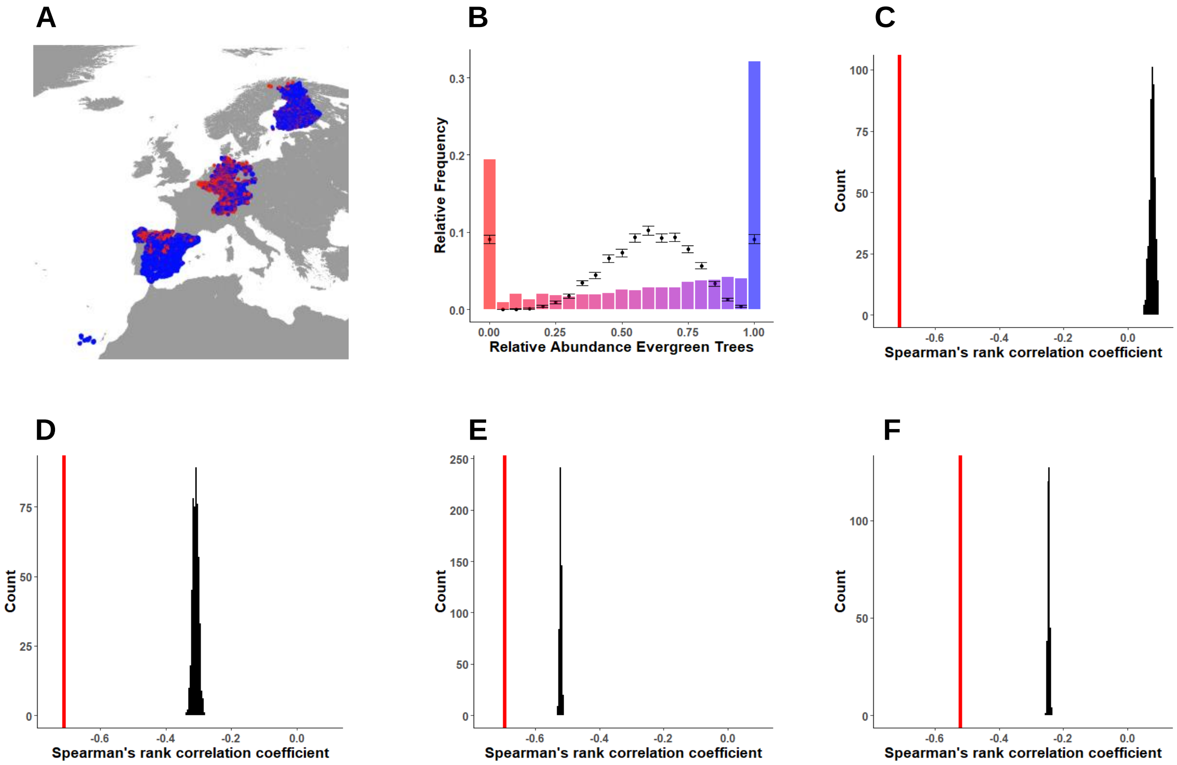

Fig. S3 | Anticorrelation between evergreen and deciduous stem density at the continental and global scale. A: The spatial distribution of over 15,431 forest inventory sites from the European NFIs used for the continental analysis. Colors represent the relative abundance of evergreen trees within a plot (red, 100% deciduous; blue, 100% evergreen). **B**: Histogram of the plot-level evergreen percentage in observed data across the Europe. The results of null model driven by environmental filtering (zero adjusted Poisson distribution) are shown as medians with 2.5% and 97.5% quantiles. **C**: Spearman's rank correlation coefficient between evergreen abundance and deciduous abundance in the observed data (the red bar) versus the simulated results of null model (the black histogram) with a Poisson assumption for European NFIs. **D**: Spearman's rank correlation coefficient between evergreen abundance and deciduous abundance in the observed data versus the simulated results of null model with a negative binomial assumption (with overdispersion correction) for European NFIs. **E**: Spearman's rank correlation coefficient between evergreen abundance and deciduous abundance in the observed data versus the simulated results of the null model with a negative binomial assumption for the US FIA data. **F**: Spearman's rank correlation coefficient between evergreen abundance and deciduous abundance in the observed data versus the simulated results of the null model with a negative binomial assumption for the GFBi dataset.

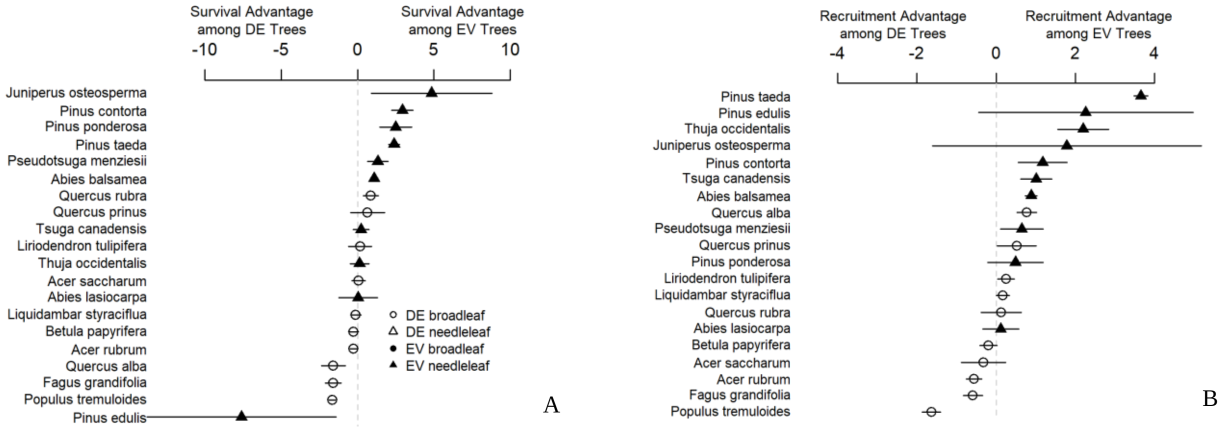

Fig. S4 | Difference in survival (A) and recruitment probability (B) of individual tree species growing in evergreen (EV)

or deciduous (DE) forests. Positive values indicate that an individual of a given tree species is more likely to survive or recruit within an evergreen forest and negative values indicate that an individual is more likely to survive or recruit within a deciduous forest. Error bars represent the 95% CI of the difference in means.

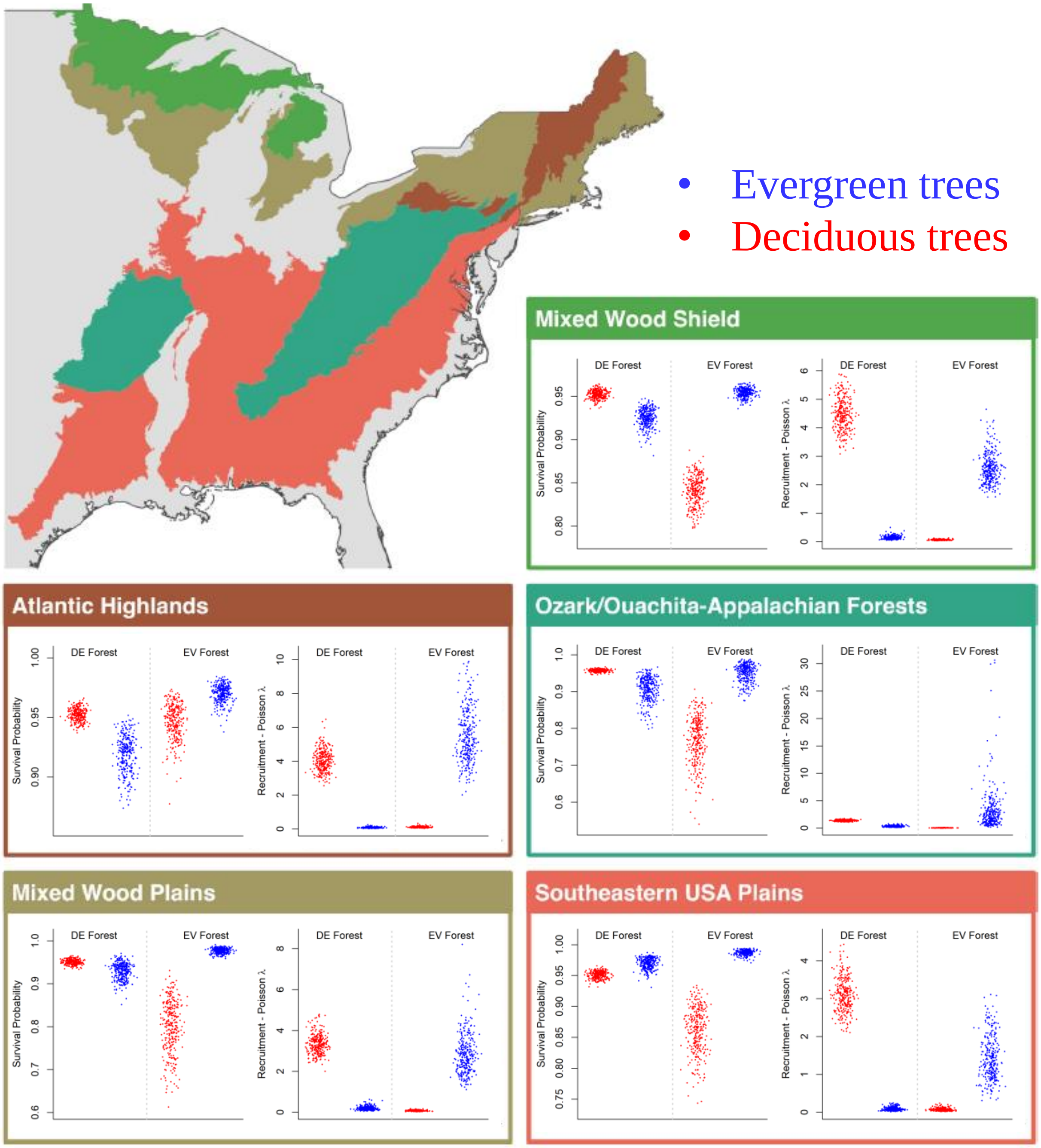

Fig. S5 | Subregional analysis of con-phenological neighborhood effects. Con-phenological feedbacks in tree recruitment and survival across 5 ecoregions in eastern US within the FIA dataset. Statistical models were fit within ecoregions that contained at least 1,000 forest inventory plots after filtering (see Methods).

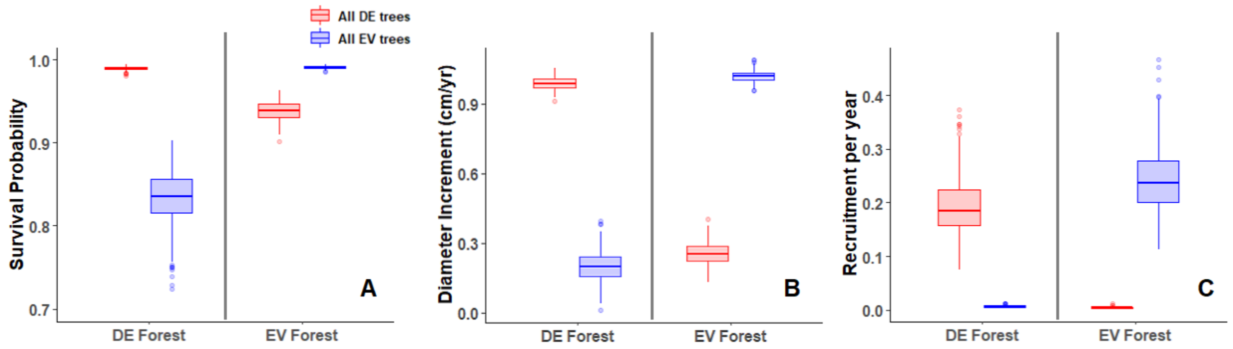

Fig. S6 | Observed positive feedback in con-phenological demographics in Europe. A: Survival probability of an individual deciduous tree (DE, red) or evergreen tree (EV, blue) within a purely evergreen or deciduous forest stand. **B**: Individual deciduous or evergreen tree growth (stem diameter increment in cm per year) when the surrounding trees are purely evergreen or deciduous. **C**: Recruitment rates of deciduous or evergreen trees in deciduous or evergreen dominated forest plots. All plotted data are drawn from the 95% CI of the corresponding full model, controlling for environmental conditions and stand structure. The differences between all compared pairs are highly significant (t-test p-value < 0.001).

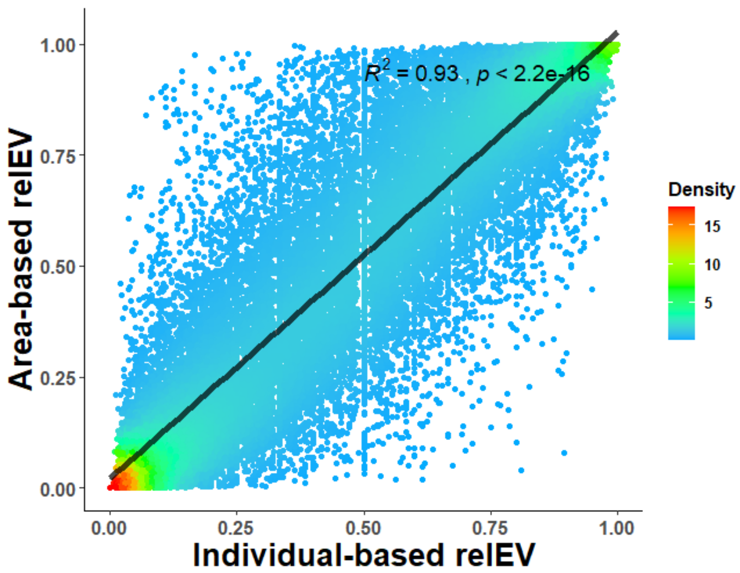

Fig. S7 | Comparison between area-based relEV (weighted by basal area) and individual-based relEV (weighted by number of stems per plots).

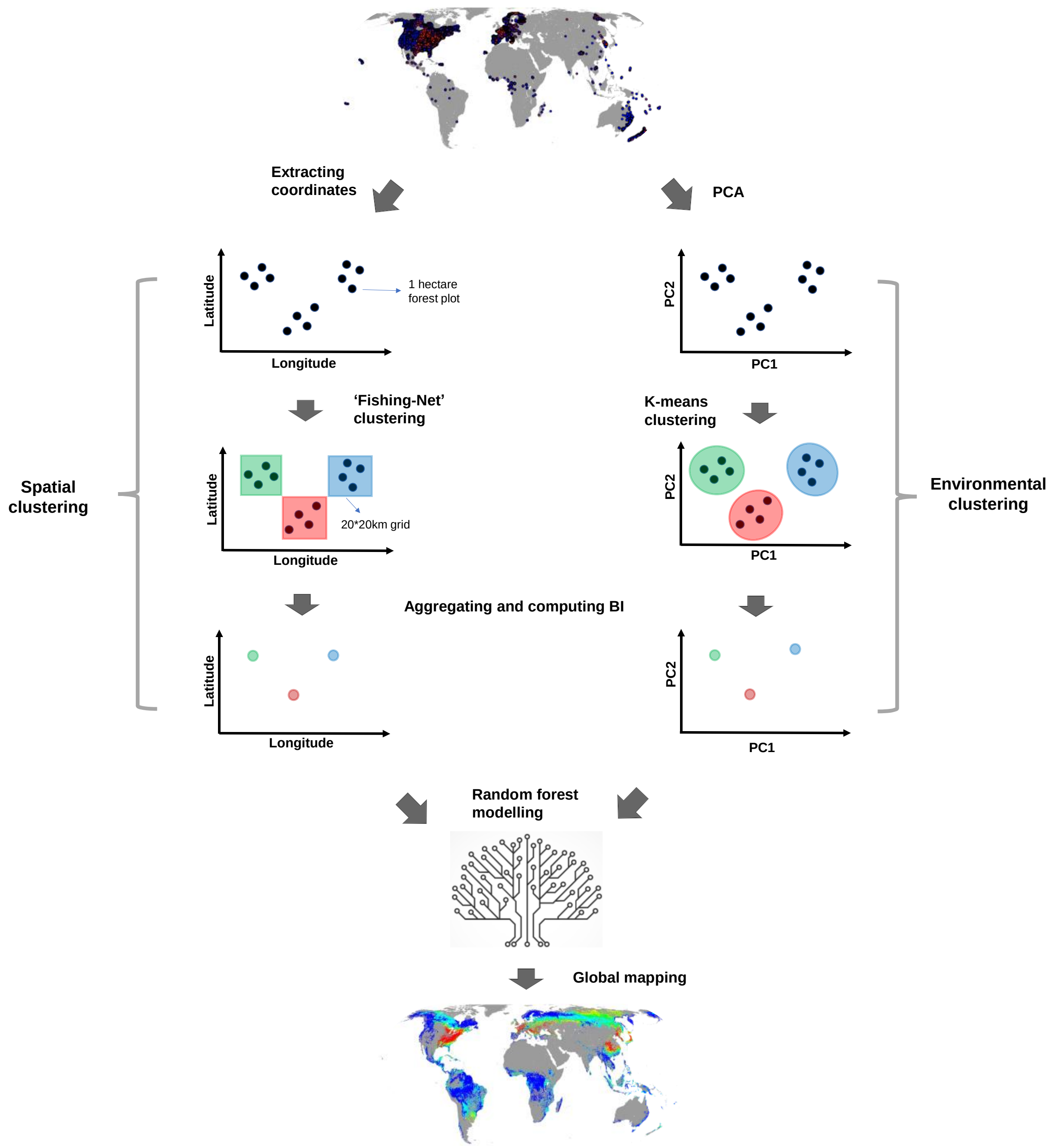

Fig. S8 | Graphic demonstration of the two independent random forest models. To generate a spatial understanding of the potential presence of alternative stable states, we developed two independent random forest models with different plot-partition methods for global projection. In the "spatial clustering" approach (left part), we partitioned the global forest zones using a 'fishing net' with 10 arc-min (~20km) grid size. For each cluster, we aggregated forest composition information of all plots to calculate a bimodality index (BI), which allowed us to quantify bimodality in the leaf phenology distribution across all plots within each cluster. The BI ranges from -1 to 1, where BIs < -0.22 represent deciduous-dominated clusters, BIs > 0.22 represent evergreen-dominated clusters, and BIs of -0.22 – 0.22 represent bimodal clusters. In the "environmental clustering" approach (right part), we implemented K-means clustering to group forest plots based on the leading three environmental PCs (for simplicity, we only show the leading two PCs in the graph) associated with each plot. Similar to the "spatial clustering" approach, for each environmental cluster, we then aggregated plot-level relative evergreen abundance to calculate the BI. To extrapolate the BI across the globe, using the "spatial clustering" and "environmental clustering" approaches, we then trained two random forest models, including the 62 environmental predictors.

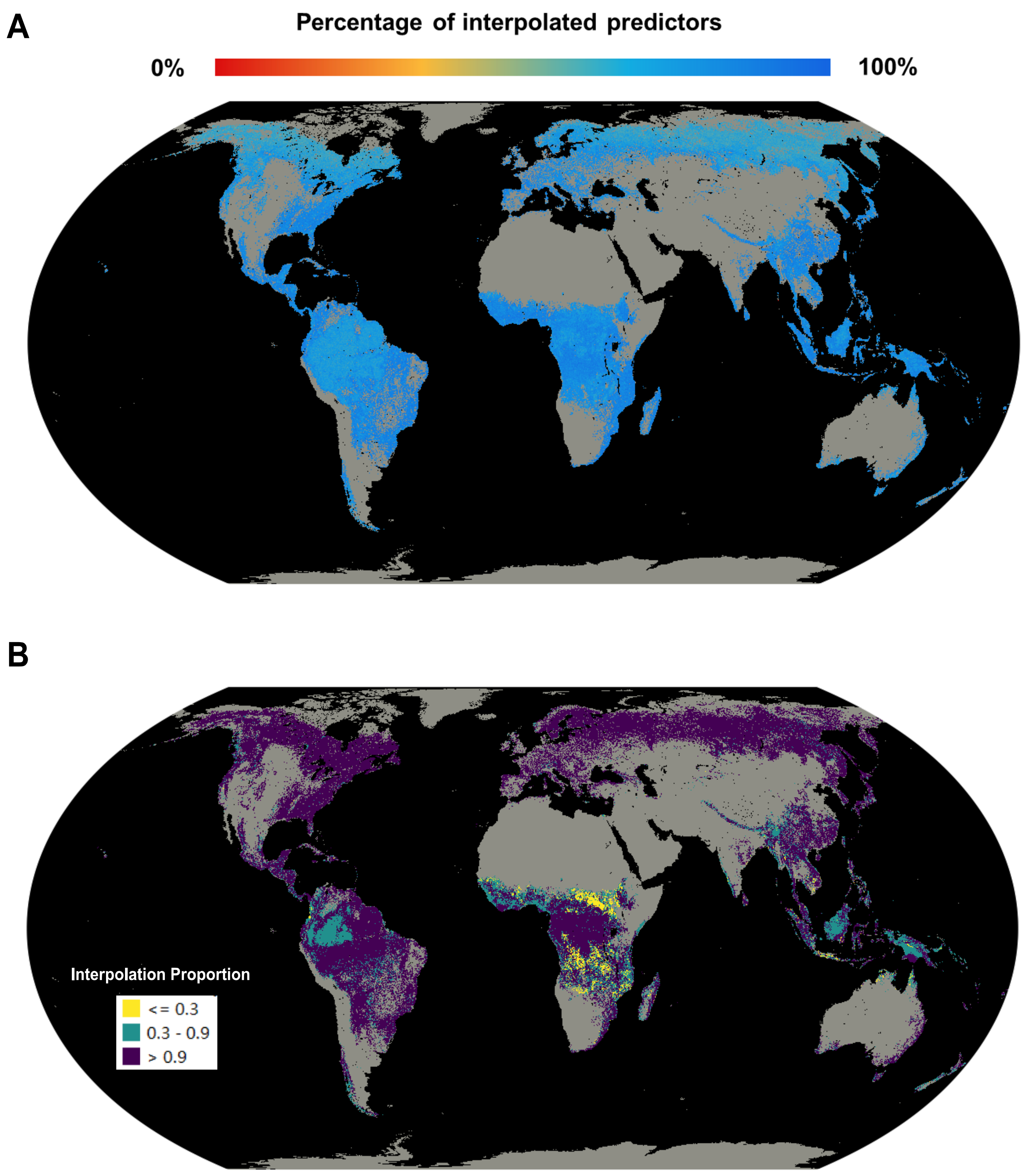
A
Percentage of interpolated predictors
0%
100%
B
Interpolation Proportion
<= 0.3
0.3 - 0.9
> 0.9

Fig. S9 | Map for interpolation vs. extrapolation analysis. A: map showing the percentage of predictors in each pixel with values falling into the range of our training dataset. B: interpolation proportion based on the convex hull methods using 66 combinations of randomly selected two PCs.

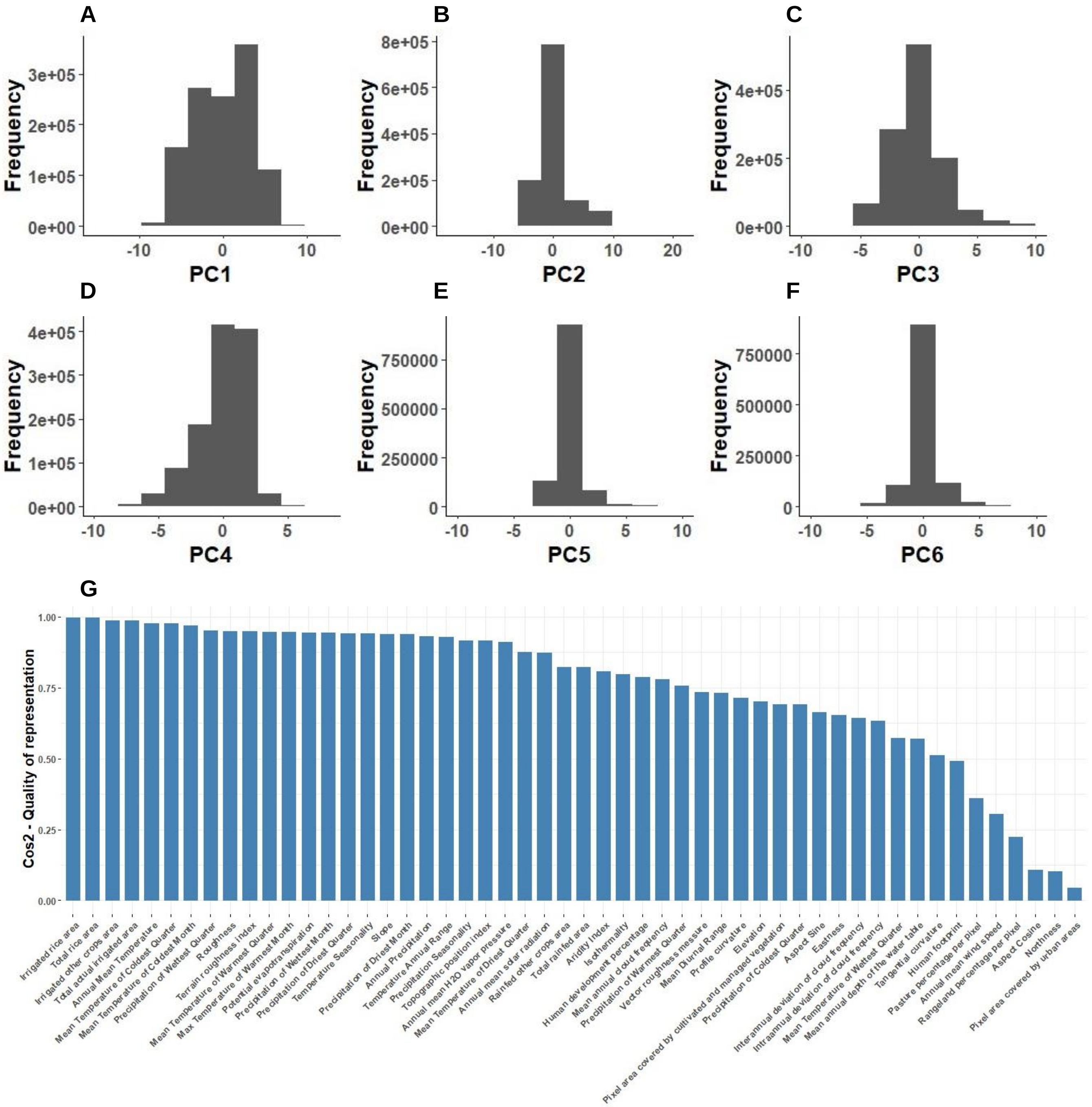

Fig. S10 | Analysis of global environmental PCA axes. A-F: histograms of the leading six PCA axes. G: Quality of

representation of the ten leading environmental PCs. For each original variable, a measure called the "cos2" (squared cosine) value is calculated. This value indicates how well that original variable is represented in the PCA plot when it is projected onto each principal component. The cos2 value ranges from 0 to 1, with 1 indicating a perfect representation of the original variable by the principal components (all the variance of the variable is represented), and 0 indicating a poor representation (none of the variance of the variable is represented). 47 of the original 53 variables are well represented (cos2 ⩾ 50%) in the ten leading PCs.

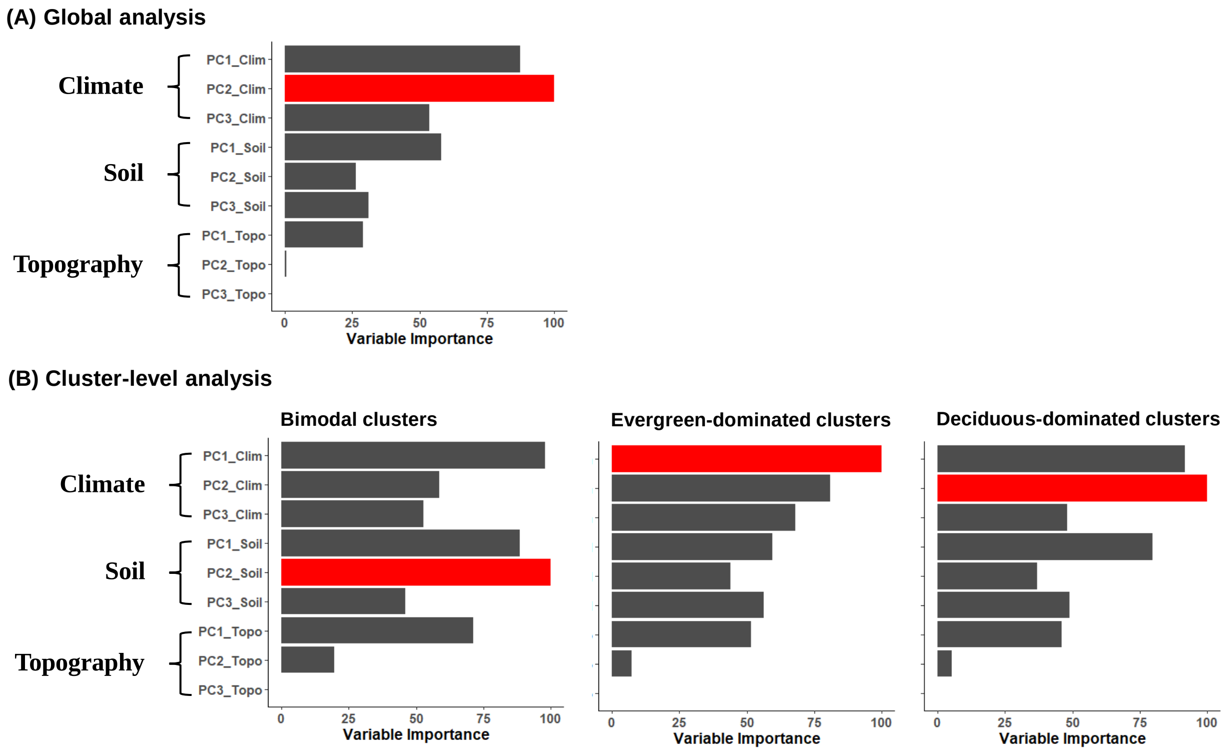

Fig. S11 | Random forest variable importance used for determinant analysis. Variables are the top three principal components of climatic, soil and topographic covariates, respectively. (A) Relative variable permutation importance from a global random forest model using BI as outcome variable. (B) Relative variable permutation importance from a random forest model using plot-level relative evergreen abundance as outcome variable and including only plots within grids with bimodal forest distribution (left panel), evergreen-dominated forest plots (middle panel) or deciduous-dominated forest plots (right panel). The most important determinants in (A) and each panel of (B) is highlighted with red color.

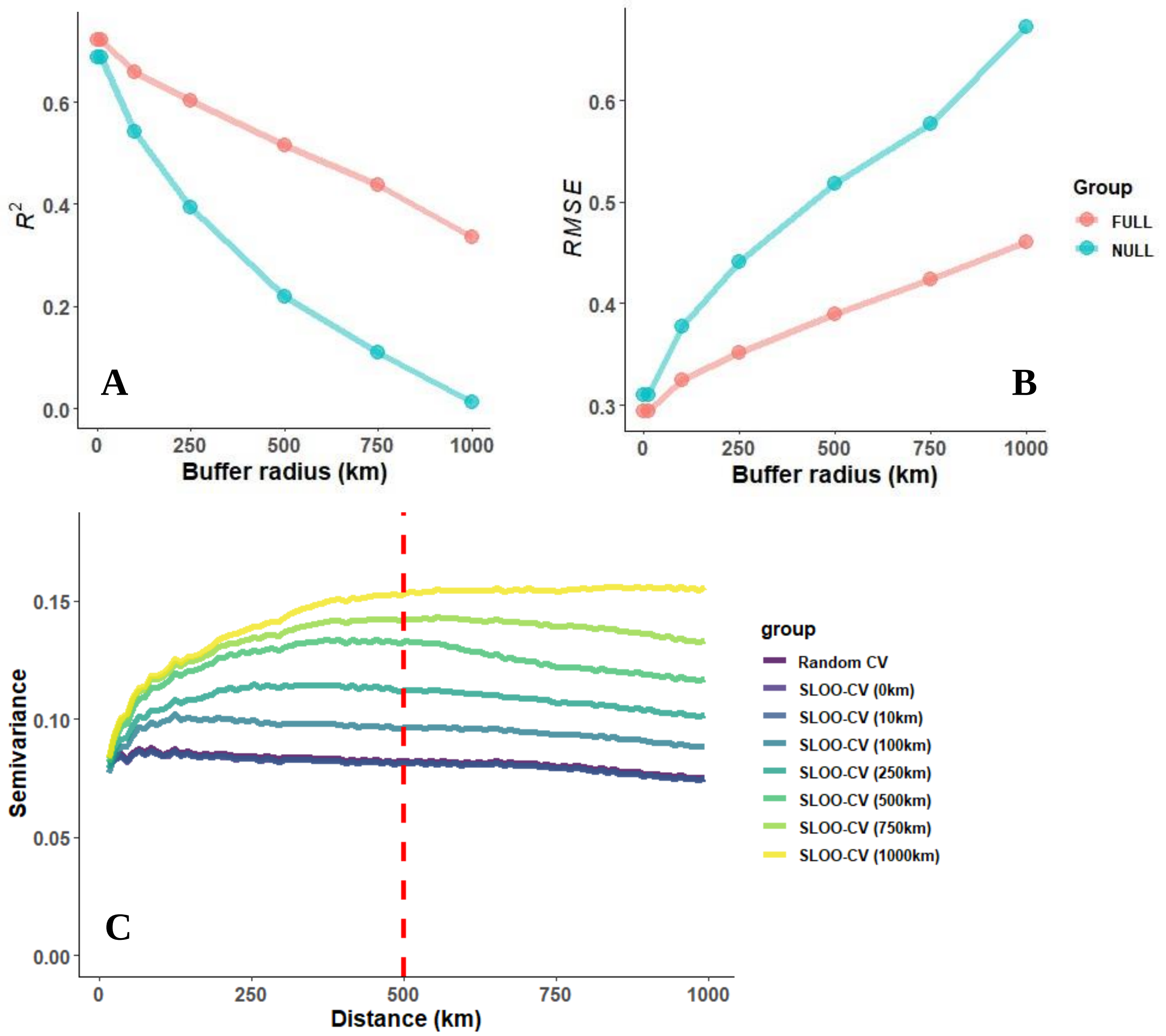

Fig. S12 | Results of spatially buffered leave-one-out analysis (SLOO-CV). (A) Coefficients of determination (R^2) for buffer radii of data exclusion from 0 km to 1000 km for both the null model (purely spatial) and the full model. (B) RMSE for buffer radii from 0 km to 1000 km for both the null model (purely spatial) and the full model. (C) Semi-variograms indicating spatial autocorrelation of model residuals for different cross-validation models. The dash line highlights the distance of 500km, above which scale the curves of semivariance become flat. This suggests there is no spatial autocorrelation beyond 500km.

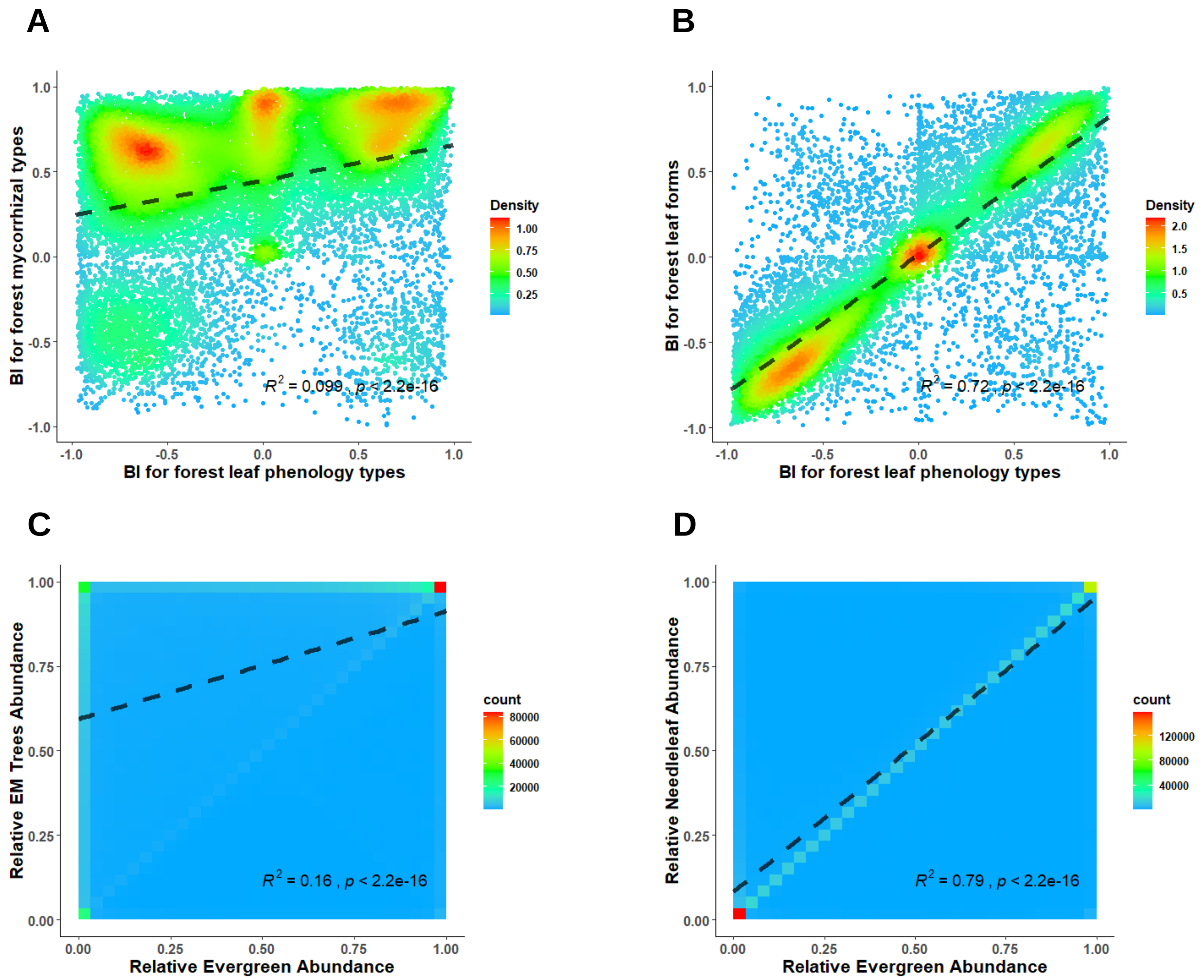

Fig. S13 | Correlation between forest leaf phenology strategies, mycorrhizal types and leaf form types. Relationship between the observed bimodality index for forest leaf phenology strategies vs. forest mycorrhizal types (A) or vs. forest leaf form types (B). Relationship between relative evergreen abundance vs. relative abundance of ectomycorrhizal-associated trees (C) or vs. relative needleleaf abundance (D).

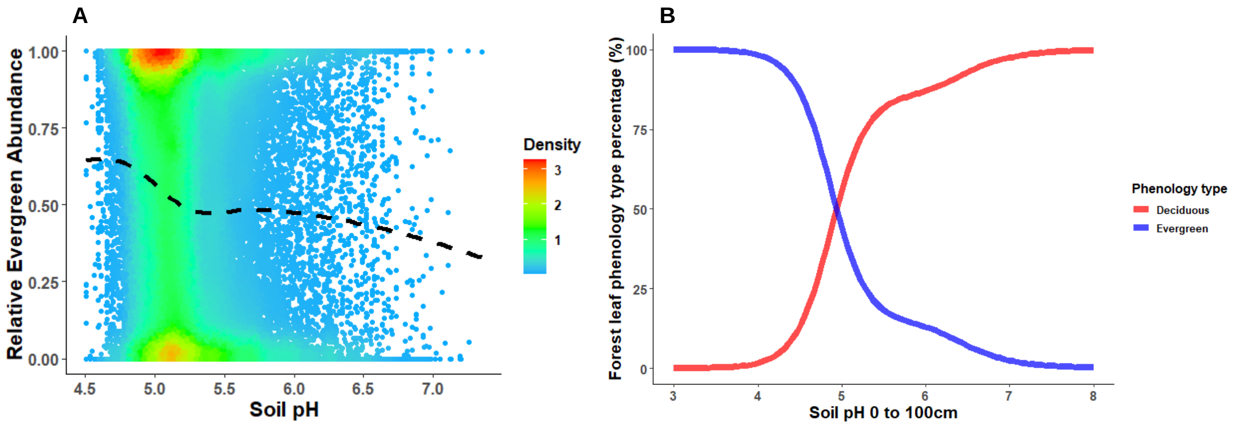

Fig. S14 | Abrupt transitions of relative evergreen abundance along gradients of soil pH in observed data (A) and the GAM model (B). A, Observed response of relative evergreen abundance to soil pH gradients. The dashed line represents a LOESS regression line. **B**, For the simulation, we first used a GAM model fitting relative evergreen abundance as a function of soil pH and other environmental covariates that cover impacts of climate, topography and soil physical properties. Then we predicted relative evergreen abundance along a gradient of changing soil pH, while keeping other variables constant. The blue line represents the predicted relative evergreen abundance, the red line represents the relative abundance of deciduous trees (1-relEV).

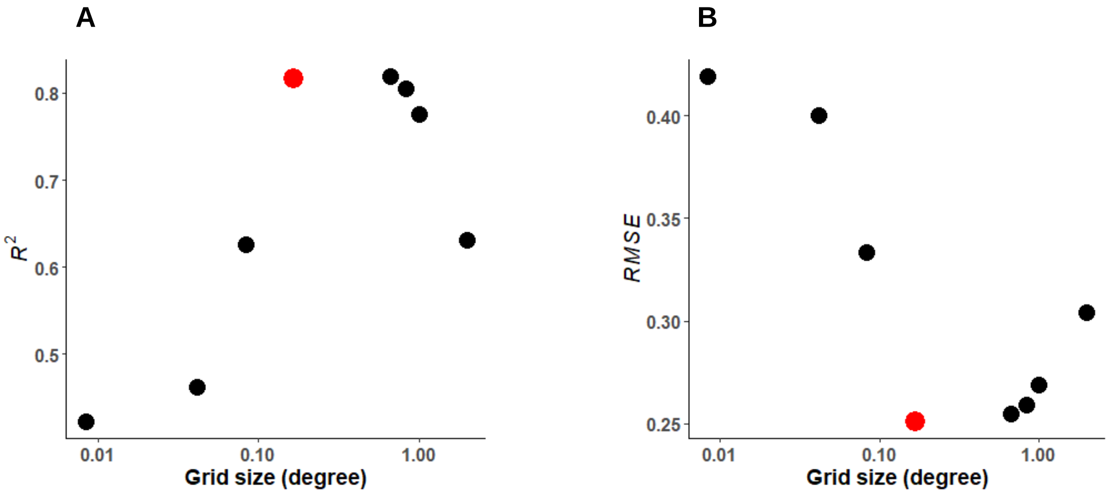

Fig. S15 | Results of Grid size tuning. To determine the optimal grid size of the "fishing net" for spatial clustering approach, we trained a series of random forest models using grid sizes from 0.01 to 2 degree. We then check R^2 (A) and RMSE (B) for each random forest model with a certain grid size.

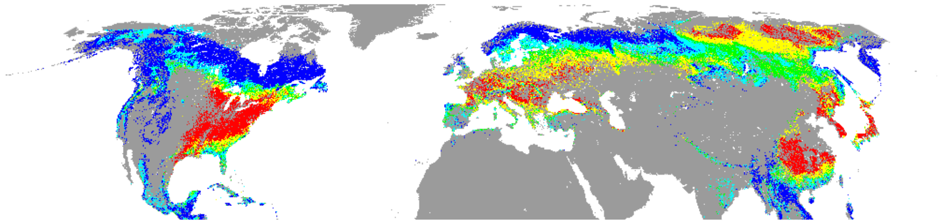

Fig. S16 | Northern Hemisphere-wide patterns of forest leaf phenology strategies. Map of bimodality in forest leaf phenology strategies based on random forest modelling using the environmental clustering approach. Colors reflect the projected value of the bimodality index (BI), with red representing BIs < -0.22 (deciduous-dominated forest clusters) and blue representing BIs > 0.22 (evergreen-dominated forest clusters), while yellow, green to cyan colors represent BIs from -0.22 – 0.22 (forest clusters with bimodal patterns). The predictions were restricted to forest regions above 15 degrees northern latitude, where 98% of the GFBi data are located.

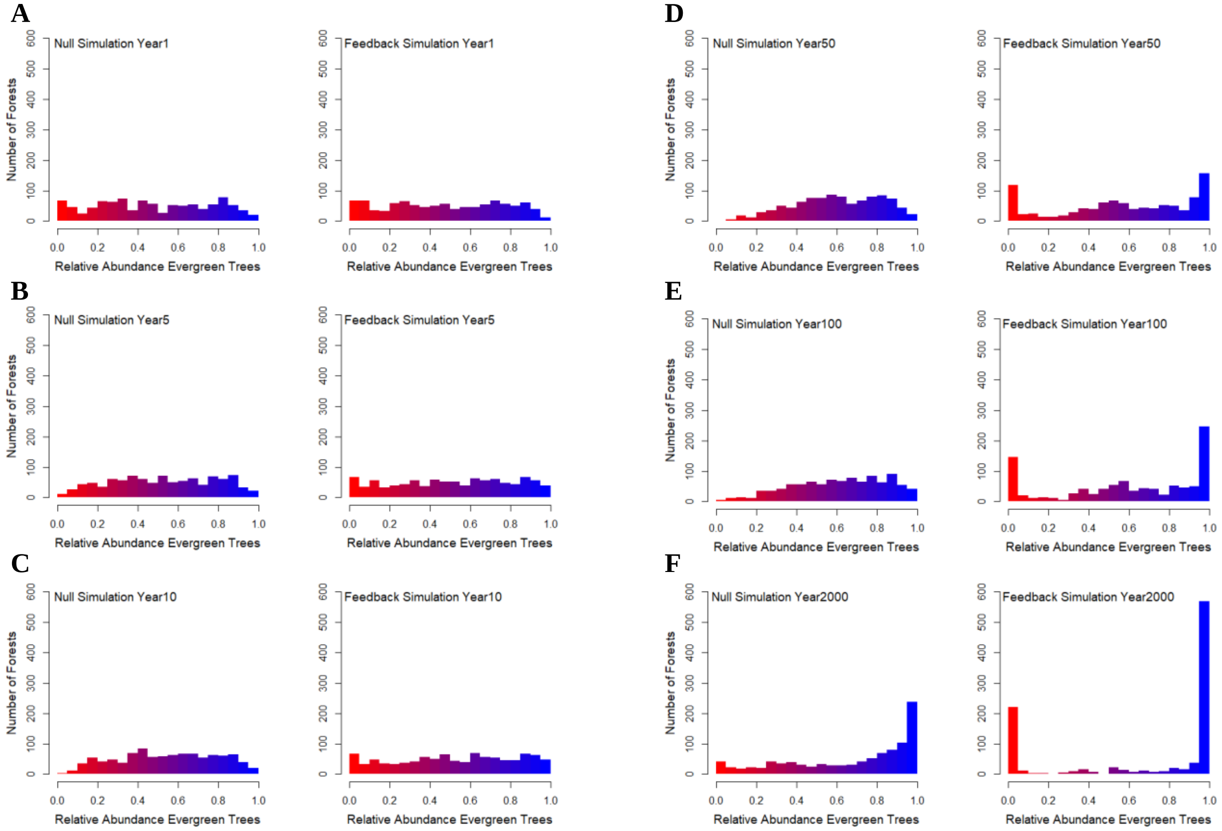

Fig. S17 | Time series of feedback simulations versus null simulations with uniform initialization. Snapshot of simulations (as shown in Fig. 3A-B) in year 1 (A), 5 (B), 10 (C), 50 (D), 100 (E) and 2000 (F). Color scale represents the percentage of evergreen forest within a plot (red, 100% deciduous; blue, 100% evergreen).

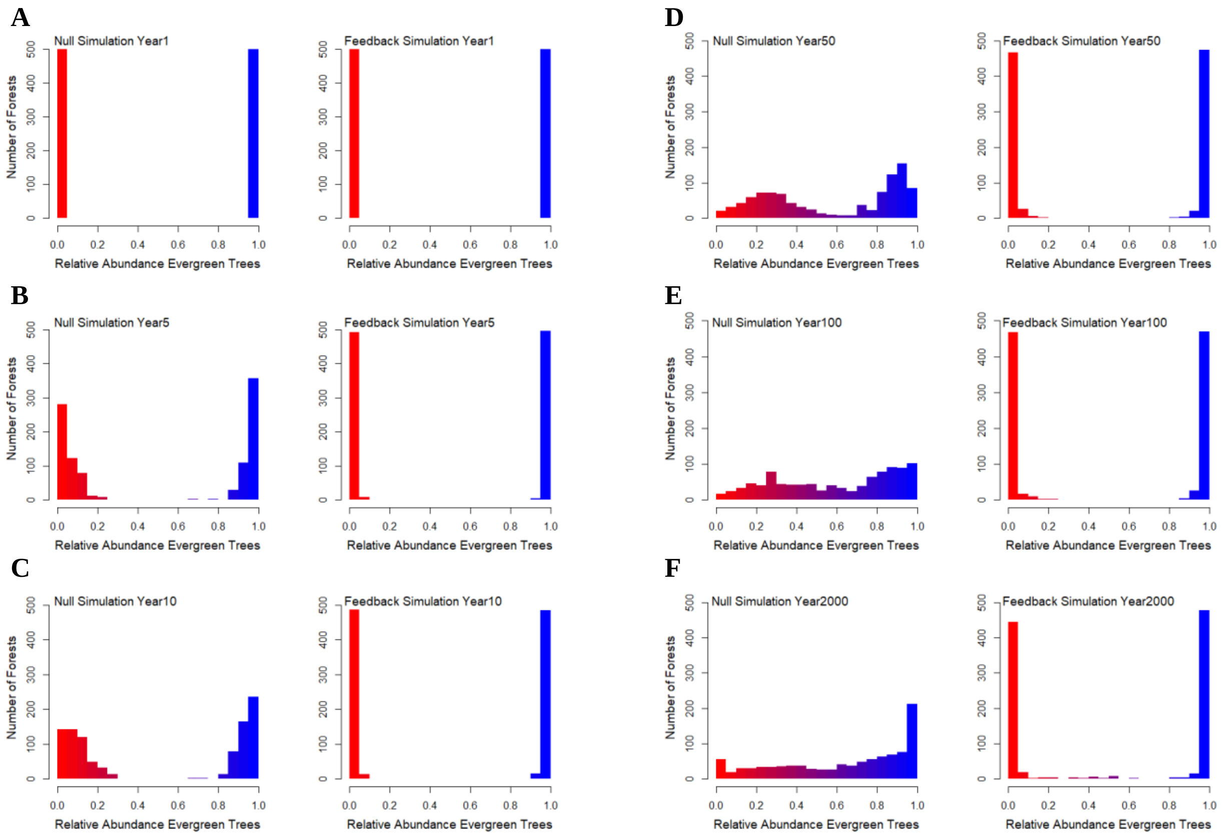

Fig. S18 | Time series of feedback simulations versus null simulations with bimodal initialization. Snapshot of simulations (as shown in Fig. 3C-D) in year 1 (A), 5 (B), 10 (C), 50 (D), 100 (E) and 2000 (F). Color scale represents the percentage of evergreen forest within a plot (red, 100% deciduous; blue, 100% evergreen).

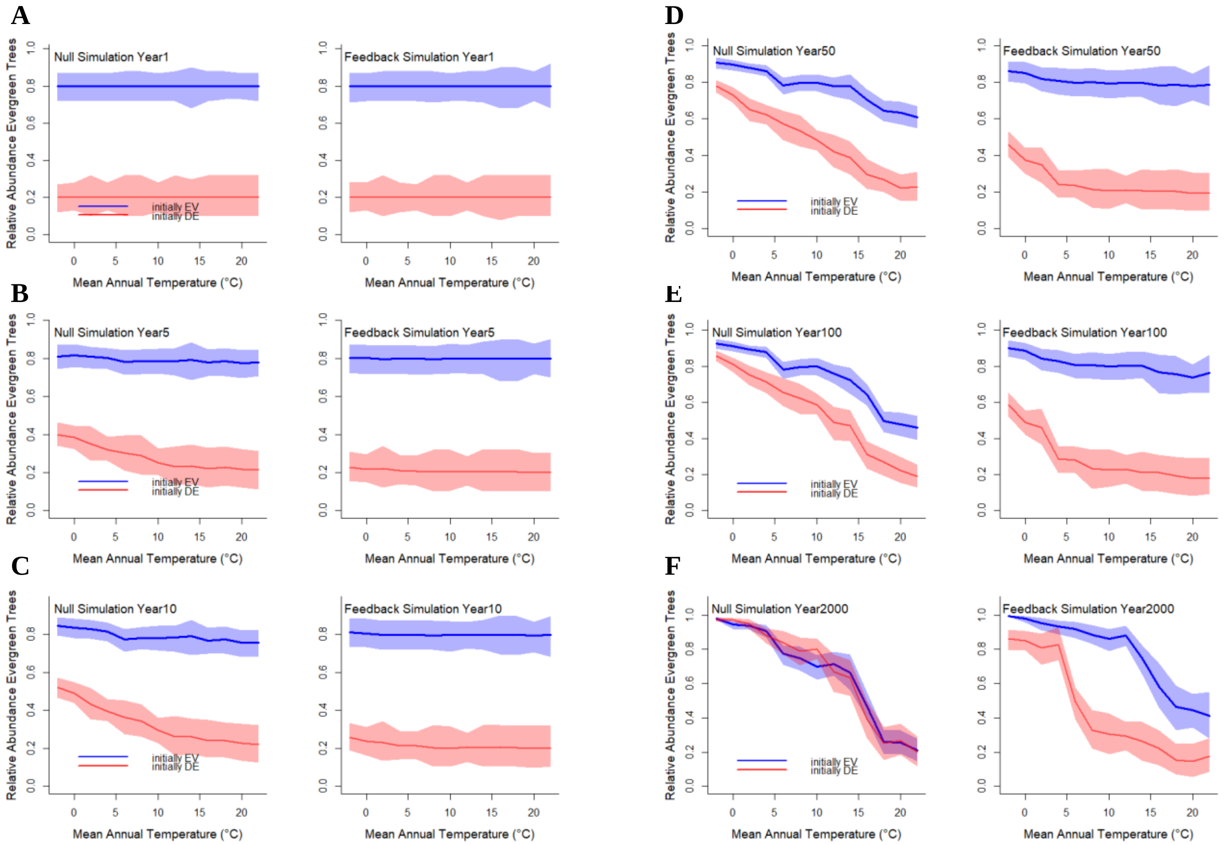

Fig. S19 | Time series of feedback simulations versus null simulations for hysteresis testing. Snapshot of simulations (as shown in Fig. 3E-F) in year 1 (A), 5 (B), 10 (C), 50 (D), 100 (E) and 2000 (F). Color scale represents the percentage of evergreen forest within a plot (red, 100% deciduous; blue, 100% evergreen).

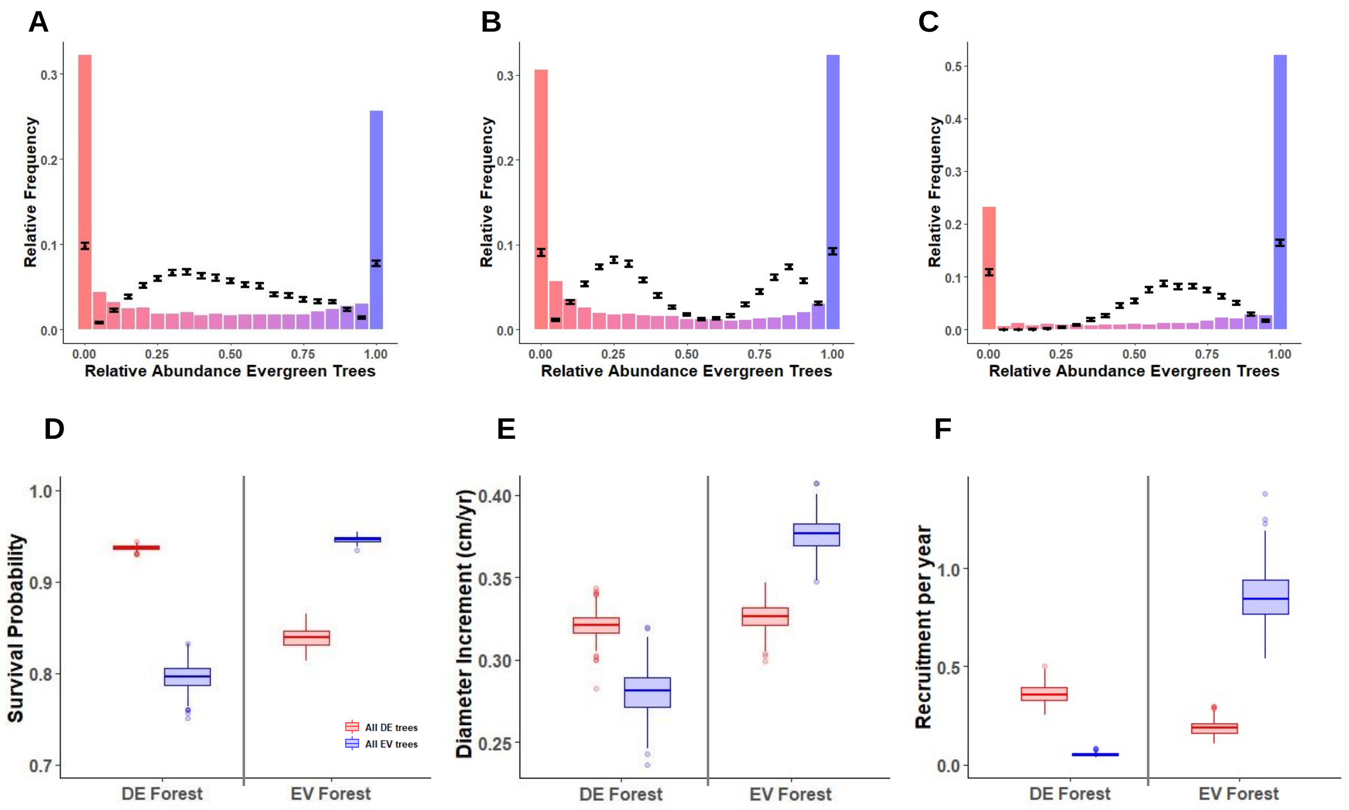

Fig. S20 | Testing the effects of successional status and monoculture. A-C: bimodality testing using forest plot data [A: GFBi, B: FIA, C: FunDivEurope] in late successional stage (plot-mean dbh > 25cm) without removing monoculture. D-E: demographic analysis using FIA data in late successional status (plot-mean dbh > 25cm) without removing monoculture.

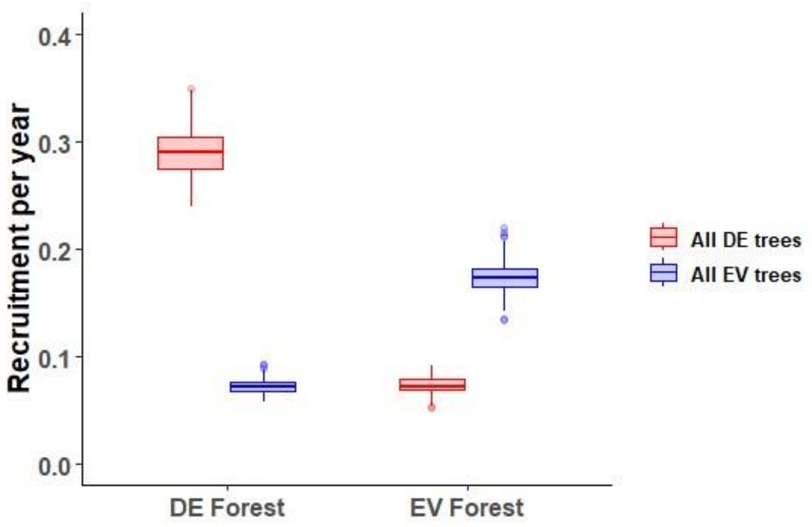

Fig. S21 | Recruitment analysis with seed source correction. Recruitment rates of deciduous (DE, red) or evergreen trees (EV, blue) in deciduous or evergreen dominated forest plots. All plotted data are drawn from the 95% CI of the corresponding full model, controlling for environmental conditions and stand structure. The differences between all compared pairs are highly significant (t-test p-value < 0.001).

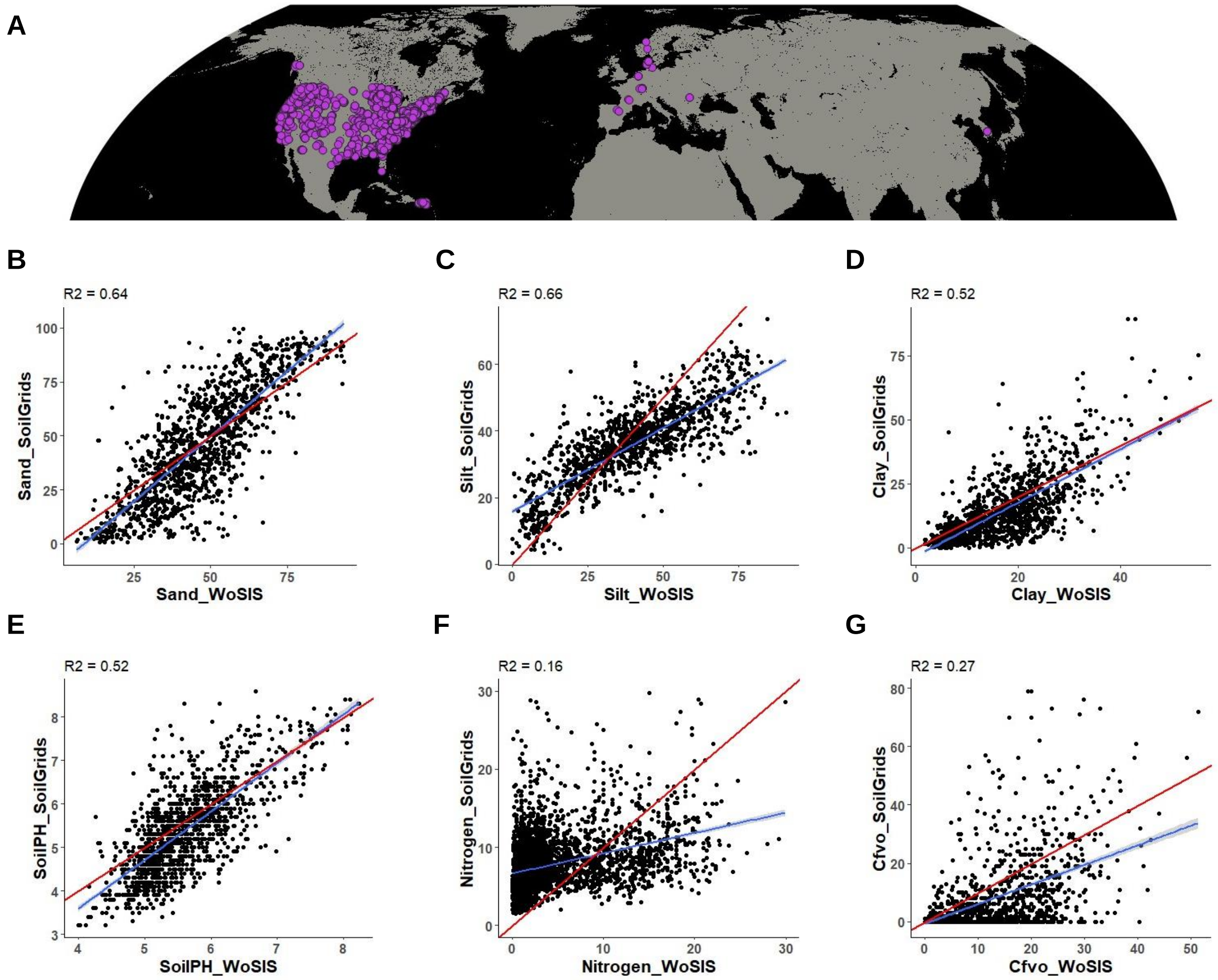

Fig. S22 | Comparison of soil data between Soil Grid and WoSIS. A: spatial distribution of WoSIS data. Purple points represent the 2346 locations with a match between the point-level WoSIS data and a forest inventory plot in GFBi dataset (see SI Section 7). B-G: Scatter plots showing the correlations of soil variables from the Soil Grids maps and the point-level WoSIS dataset. The correlations were evaluated for six variables, which were also used for random forest modelling: soil sand content (B, mass fraction in %), soil silt content (C, mass fraction in %), clay content (D, mass fraction in %), soil pH (E), soil nitrogen density (F, g/kg) and soil coarse fragments volumetric (G, mass fraction in %).

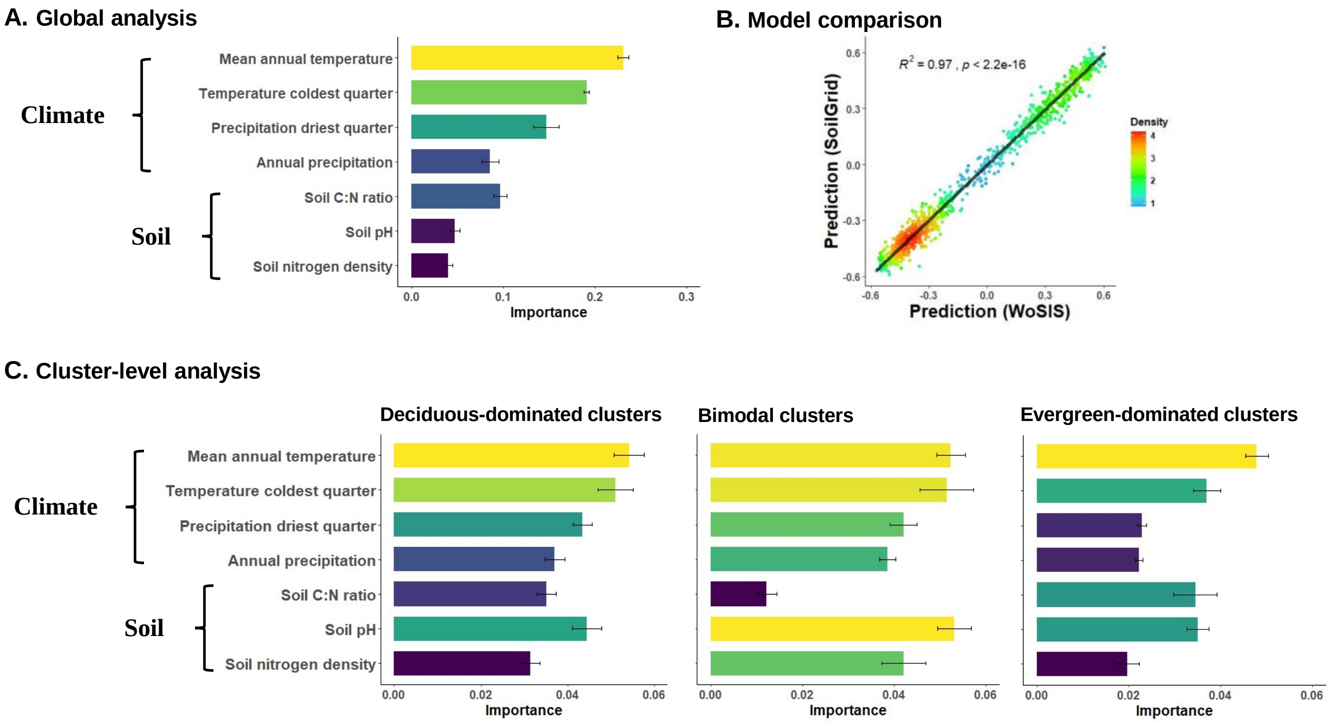

Fig. S23 | Random forest analysis using point-level soil data (WoSIS within 1000m radius around GFBi plot). Panel A and C depict determinant analyses akin to Fig. 5, with the incorporation of WoSIS data for soil pH and soil nitrogen density. Panel B presents a scatter plot, demonstrating the correlation of predictions following a ten-fold cross-validation process between two random forest models trained on WoSIS and Soil Grids, respectively. Both models anticipate the bimodality index using a set of 62 predictors (Table. S2), which comprise 9 soil covariates and 53 covariates that encapsulate climate, human impact, and topography. In the first random forest model, six out of the nine soil covariates derive from Soil Grids. However, in the second model, these six variables are sourced from WoSIS.

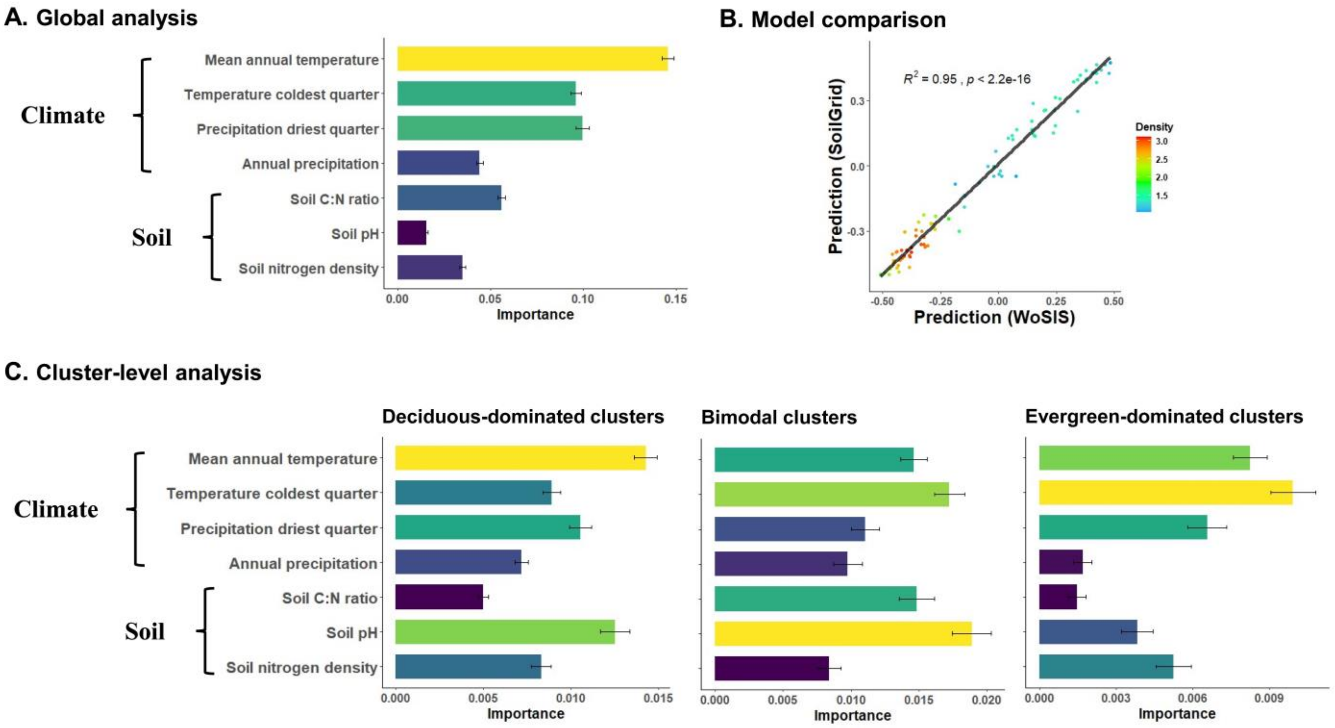

Fig. S24 | Random forest analysis using point-level soil data (WoSIS within 250m radius around GFBi plot). Panel A and C depict determinant analyses akin to Fig. 5, with the incorporation of WoSIS data for soil pH and soil nitrogen density. Panel B presents a scatter plot, demonstrating the correlation of predictions following a ten-fold cross-validation process between two random forest models trained on WoSIS and Soil Grids, respectively. Both models anticipate the bimodality index using a set of 62 predictors (Table. S2), which comprise 9 soil covariates and 53 covariates that encapsulate climate, human impact, and topography. In the first random forest model, six out of the nine soil covariates derive from Soil Grids. However, in the second model, these six variables are sourced from WoSIS.

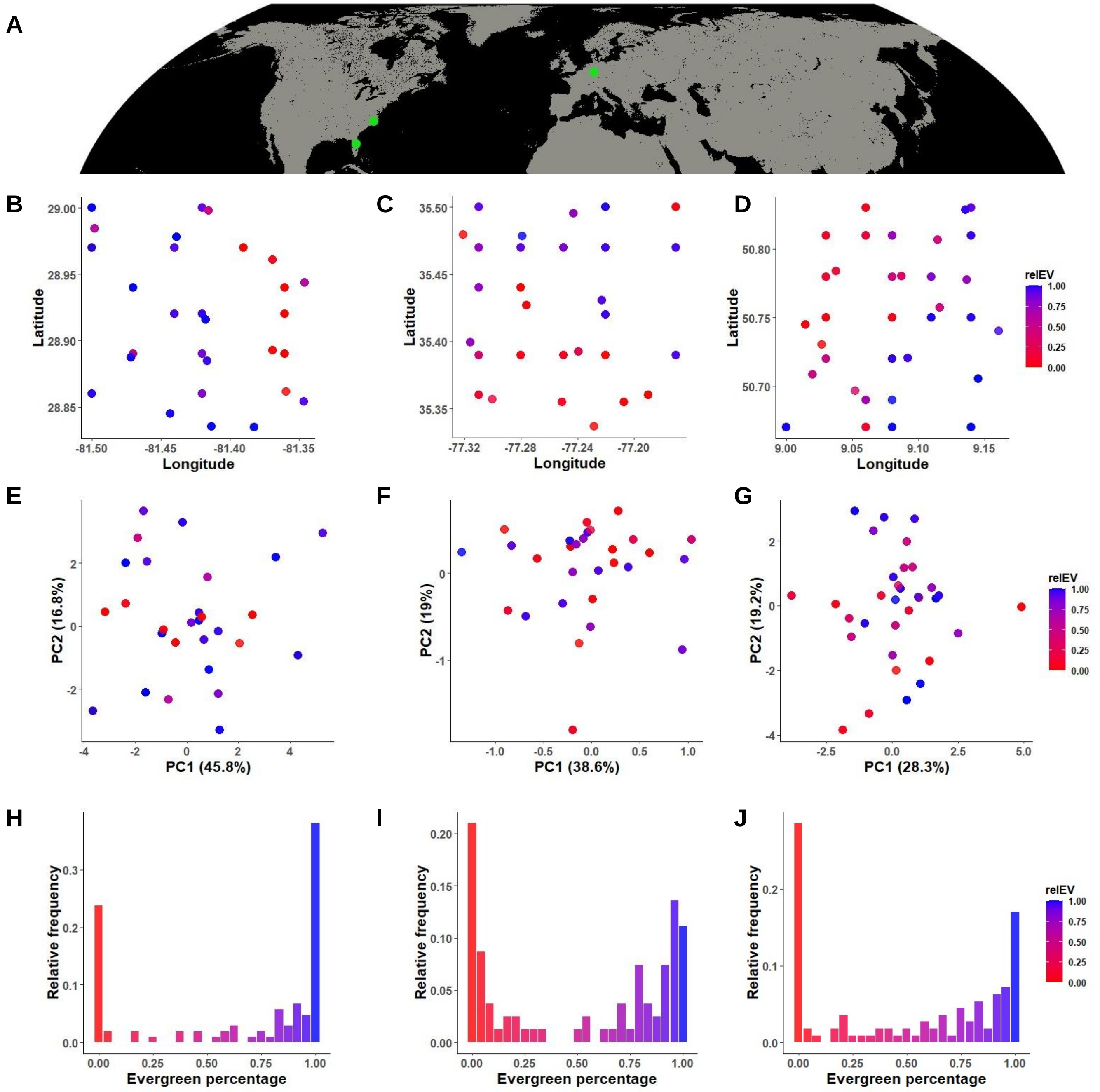

Fig. S25 | Distribution of forest plots in example bimodal clusters. A: locations of three example 20km*20km bimodal clusters (in green). B-D: spatial distribution of forest plots in the corresponding clusters from left to right, with colors indicating plot-level relative evergreen abundance. E-G: locations of forest plots in PCA space spanned by the leading two PCs, for each bimodal cluster from left to right. H-J: histograms of the observed plot-level relative evergreen abundance in each cluster from left to right.

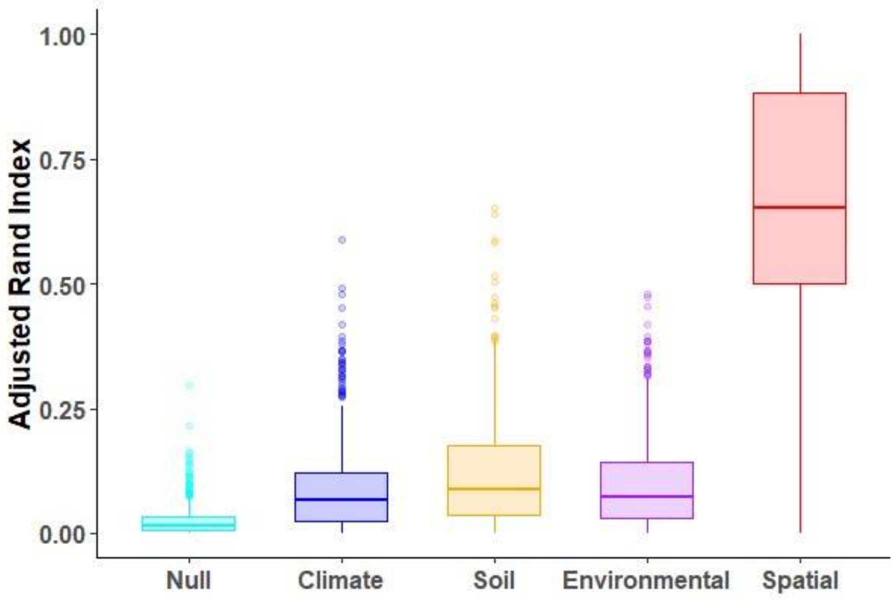

Fig. S26 | Comparison between forest phenological status and five types of grouping. To assess the alignment of the five types of groupings (spatial, environmental, climate, soil and random) with our predetermined classes based on forest phenological composition, we used the Adjusted Rand Index (ARI). The distribution of ARI is shown for the five types of groupings: random (Null) in cyan, climate in blue, soil in orange, environmental in purple and spatial in red.

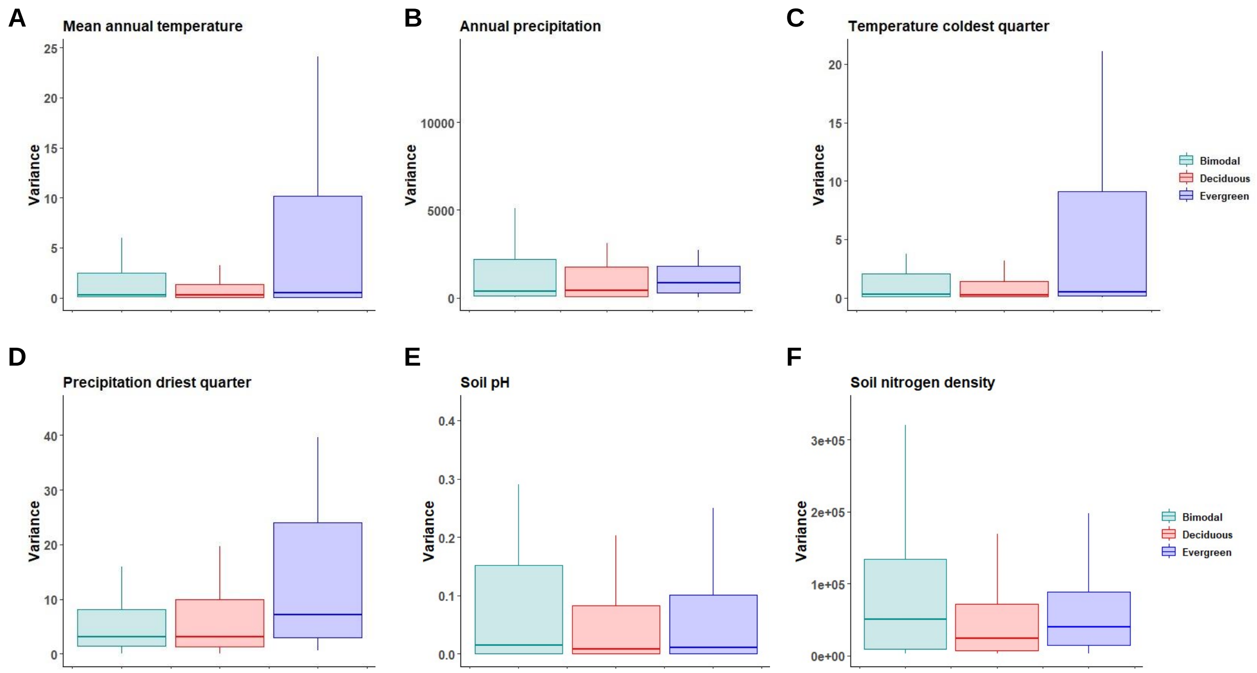

Fig. S27 | Environmental variance comparison among bimodal, deciduous-dominated and evergreen-dominated clusters. Environmental variance within bimodal clusters is shown in green, while environmental variance among plots randomly selected from the entire GFBi dataset is shown in purple.

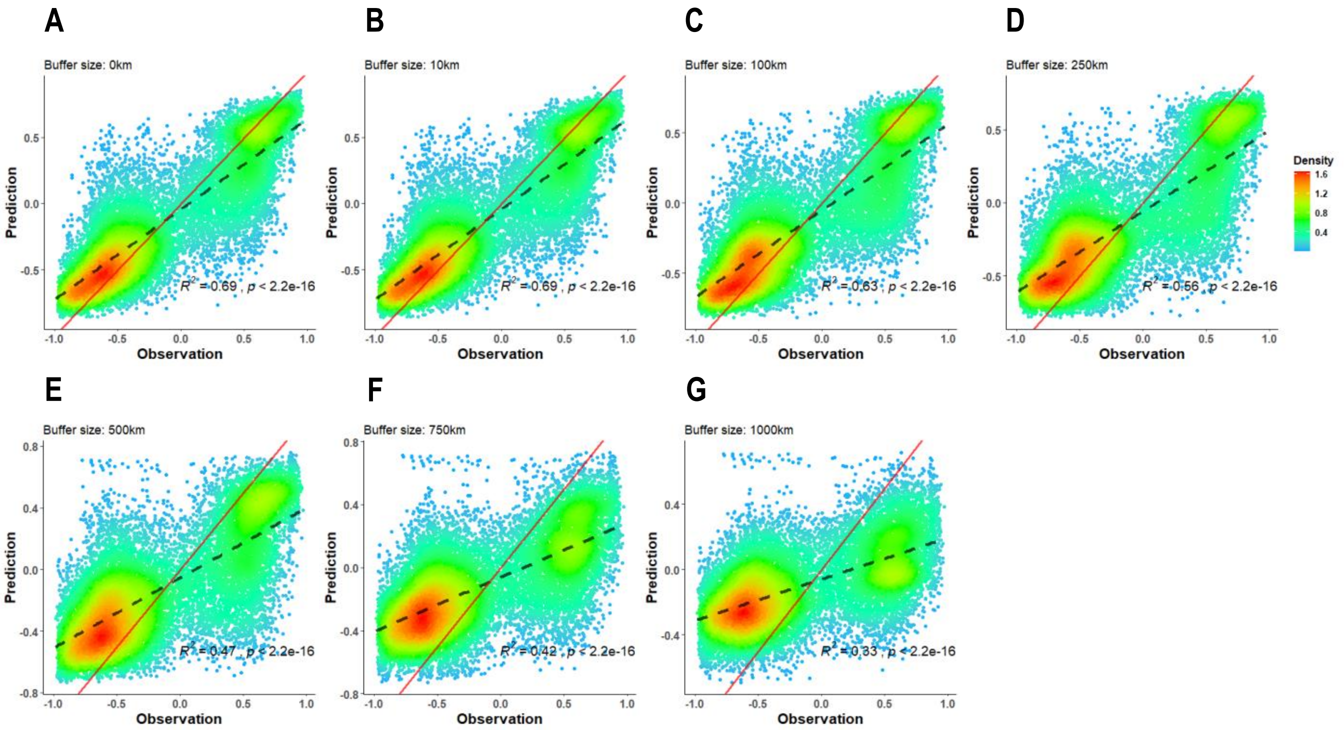

Fig. S28 | Scatter-plots showing the predictions of the full model versus observations across buffer raii (0-100km, A-G). The black-dash lines are the fitted linear regression line. While the red solid lines represent the "y=x" line.

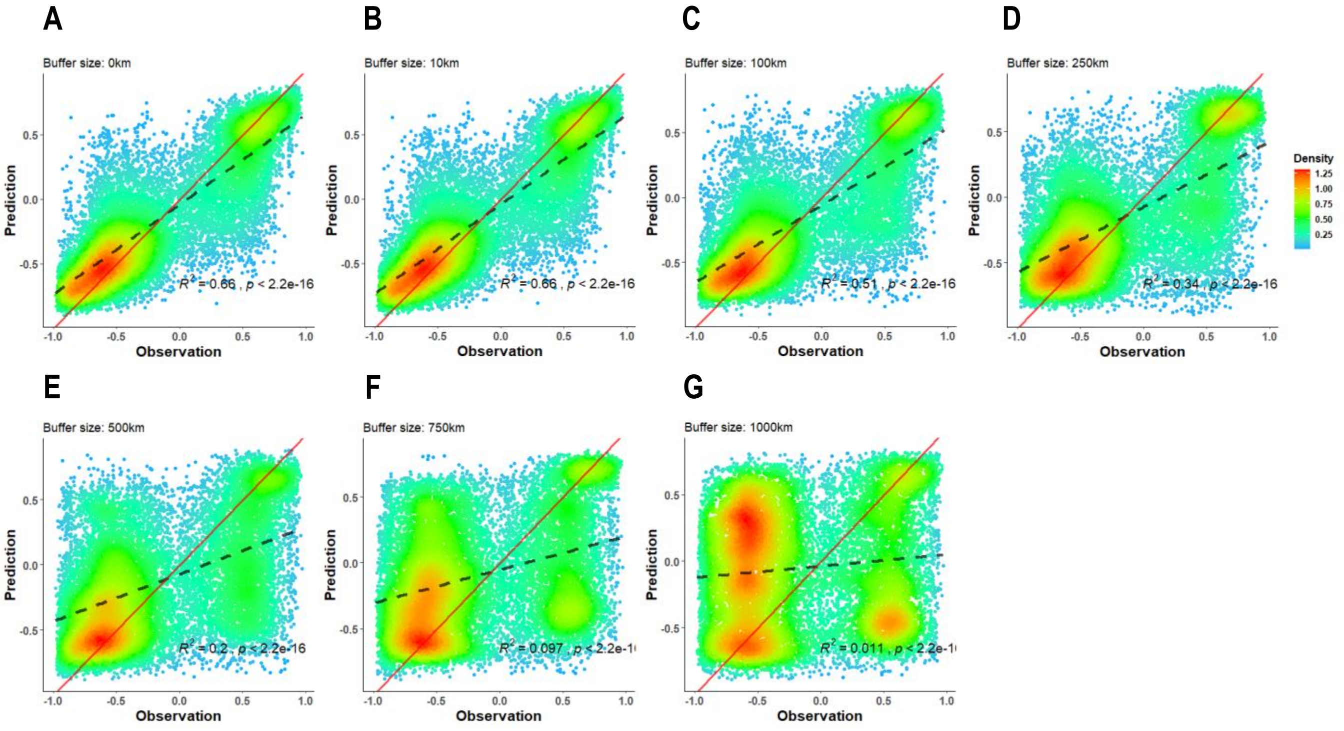

Fig. S29 | Scatter-plots showing prediction of the null model (purely spatial) versus observation across buffer raii (0-100km, A-G). The black-dash lines are the fitted linear regression line. While the red solid lines represent the "y=x" line.

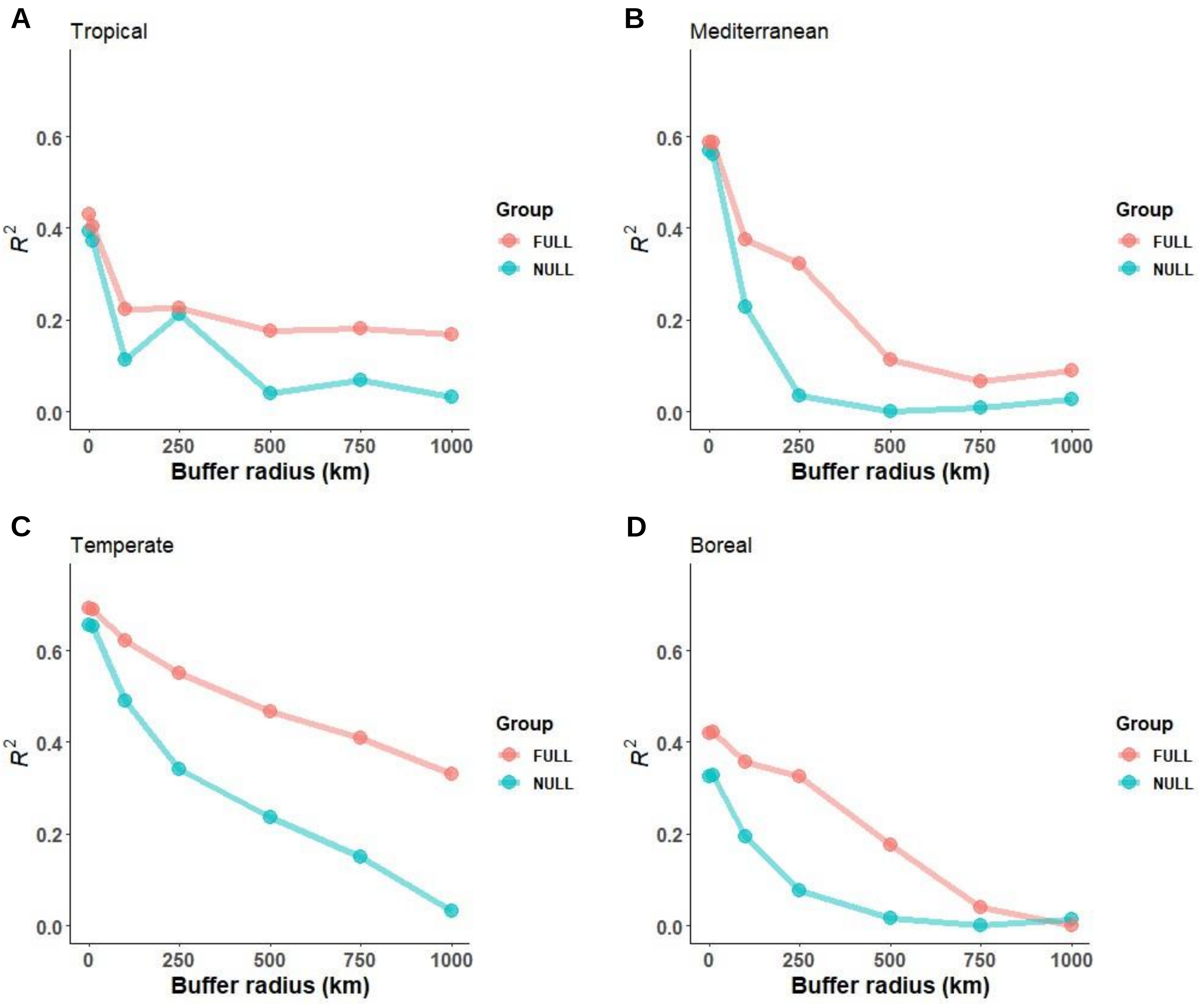

Fig. S30 | Spatially buffered leave-one-out cross validation (SLOO-CV) across biomes. Coefficients of determination (R2) for buffer radii of data exclusion from 0 km to 1000 km for both the null model (purely spatial) and the full model for tropical (A), mediterranean (B), temperate (C) and boreal forests (D).

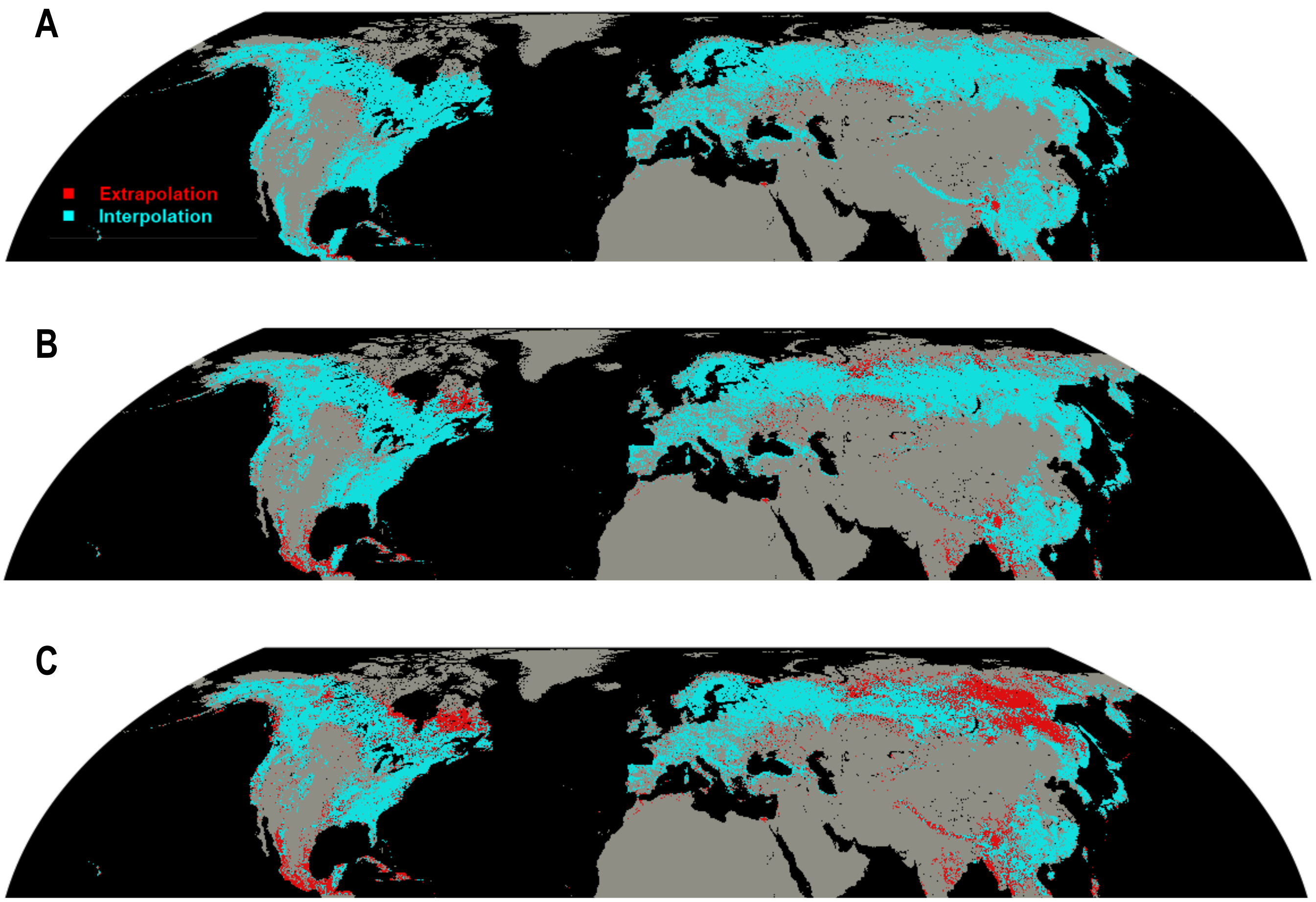

Fig. S31 | Interpolation vs. extrapolation maps based on convex hulls generated from the leading two (A), three (B) or four (C) PCs.

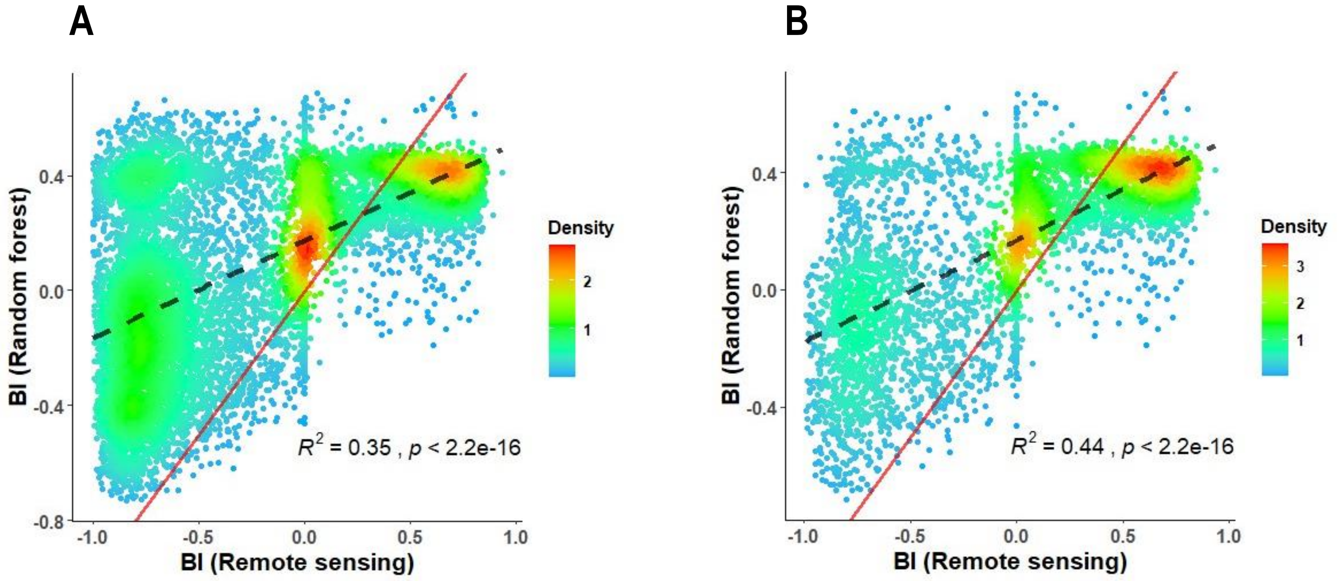

Fig. S32 | Scatter plot showing model predictions versus remote sensing observations for European forests before and after filtering. The filtering process removed all broadleaf evergreen and needle-leaf deciduous forests, and only retained needle-leaf evergreen and broadleaf deciduous trees, allowing for comparisons between model predictions and

observations.

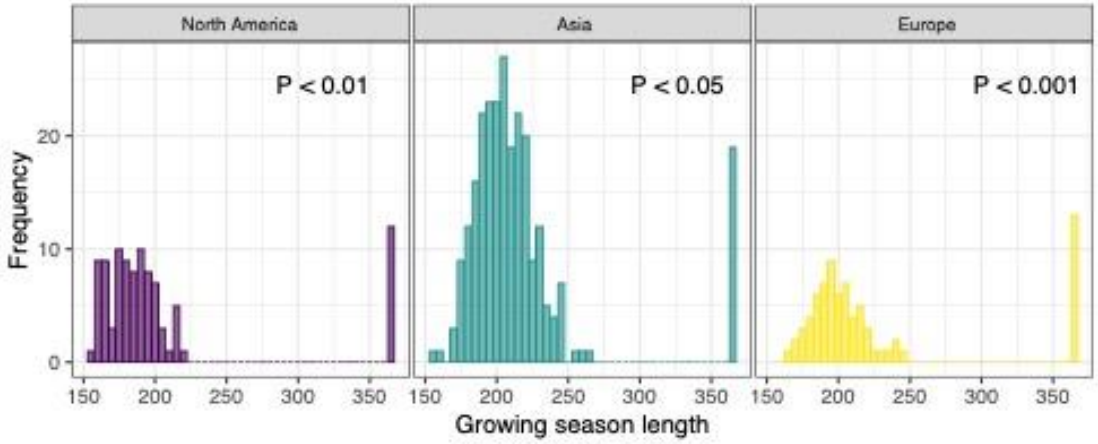

Fig. S33 | Bimodal patterns in deciduousness when treating leaf shedding as a continuous trait. To demonstrate that the distribution of growing season length is bimodal, we used species-level data from Zohner et al. (2017), which includes data for more than 400 species from North America, Europe and Asia[11]. Across all continents, there is a clear bimodal distribution of evergreen (growing season length = 365 days) and deciduous species (growing season length < 275 days), which was statistically confirmed (P <0.05) by a Hartigan's dip test.

Supplementary Tables

Table. S1 Contingency table for testing the correlation between leaf phenology strategies and mycorrhizal types of 711 tree species in the mainland US.

Mycorrhiza / Leaf Phenology	Ectomycorrhizal	Arbuscular mycorrhizal
**Evergreen**	77 (count of species)	389
**Deciduous**	67	178

Table. S2 Information on the 62 selected covariate layers used to map the forest bimodality index.

Variable	Variable group	Original spatial resolution	Units	Source
Global AI (Aridity Index)	Climatic	≈1km	–	Karger et al.[13]
Annual Mean Temperature			ºC * 10	

Annual Precipitation			mm	
Isothermality			–	
Max Temperature of Warmest Month			°C	
Mean Diurnal Range			°C	
Mean Temperature of Coldest Quarter			°C	
Mean Temperature of Driest Quarter			°C	
Mean Temperature of Warmest Quarter			°C	
Mean Temperature of Wettest Quarter			°C	
Mean Temperature of Coldest Month			°C	
Precipitation of Coldest Quarter			mm	
Precipitation of Driest Month			mm	
Precipitation of Driest Quarter			mm	
Precipitation of Warmest Quarter			mm	
Precipitation of Wettest Month			mm	
Precipitation of Wettest Quarter			mm	
Precipitation Seasonality			mm	
Temperature Annual Range			°C	
Temperature Seasonality			°C	
Mean annual cloud frequency			% of cloudy days	Wilson & Jetz[14]
Interannual deviation of cloud frequency				
Intraannual deviation of cloud frequency				
Annual mean solar radiation		≈1km	KJ/(m^2*day)	Fick & Hijmans[15]
Annual mean wind speed		≈1km	m/s	
Annual mean H2O vapor pressure		≈1km	kPa	
Elevation (in meters)	Topography	≈1km	m	Amatulli et al.[16]
Aspect Cosine			-	
Aspect Sine			-	
Eastness			-	
Northness			-	
Profile curvature			-	
Tangential curvature			-	
Terrain roughness index			-	
Vector roughness messure			-	
Topographic position index			-	
Roughness			–	
Slope			–	
Human footprint in 2009	Human	≈1km	-	Venter et al.[17]

Human development percentage		≈1km	%	Tuanmu & Jetz[18]
Pixel area covered by cultivated and managed vegetation		≈1km	%	
Pixel area covered by urban areas		≈1km	%	
Irrigated rice area		≈1km	km^2	Goldewijk[19]
Irrigated other crops area		≈1km	km^2	
Rainfed rice area		≈1km	km^2	
Rainfed other crops area		≈1km	km^2	
Total actual irrigated area		≈1km	km^2	
Total rainfed area		≈1km	km^2	
Total rice area		≈1km	km^2	
Mean annual depth of the water table on the terrestrial land surface (in m below land surface)	Geological	≈1km	m	Fan et al.[20]
Absolute Depth to Bedrock	Soil	≈250m	cm	Hengl et al.[21]
Soil clay content (0–2 micrometer) at 0 to 100cm			%	
Soil coarse fragments volumetric at 0 to 100cm			%	
Soil sand content (50–2000 micrometer) at 0 to 100cm			%	
Soil silt content (2–50 micro meter) at 0 to 100cm			%	
Soil nitrogen density at 0 to 100cm			cg/kg	
Soil pH in H2O at 0 to 100cm			pH	
Soil moisture profile		≈10km	%	Entekhabi et al.[22]
Soil C:N ratio at 0 to 100cm		≈1km	–	Batjes et al.[23]
Rangeland percentage per pixel	Process	≈1km	%	Goldewijk et al.[24]
Pasture percentage per pixel			%	
Potential evapotranspiration		≈1km	mm	Zomer et al.[25]

Supplementary References

1 Stoklosa, J., Blakey, R. V. & Hui, F. K. C. An Overview of Modern Applications of Negative Binomial Modelling in Ecology and Biodiversity. *Diversity* **14** (2022). https://doi.org:10.3390/d14050320

2 Martin, T. G. *et al.* Zero tolerance ecology: improving ecological inference by modelling the source of zero observations. *Ecol Lett* **8**, 1235-1246 (2005). https://doi.org:https://doi.org/10.1111/j.1461-0248.2005.00826.x

3 Omernik, J. M. & Griffith, G. E. Ecoregions of the Conterminous United States: Evolution of a Hierarchical Spatial Framework. *Environmental Management* **54**, 1249-1266 (2014). https://doi.org:10.1007/s00267-014-0364-1

4 van den Hoogen, J. *et al.* A geospatial mapping pipeline for ecologists. *bioRxiv*, 2021.2007.2007.451145 (2021). https://doi.org:10.1101/2021.07.07.451145
5 Ma, H. *et al.* The global distribution and environmental drivers of aboveground versus belowground plant biomass. *Nature Ecology & Evolution* **5**, 1110-1122 (2021). https://doi.org:10.1038/s41559-021-01485-1
6 Bruelheide, H. *et al.* Community assembly during secondary forest succession in a Chinese subtropical forest. *Ecol Monogr* **81**, 25-41 (2011). https://doi.org:https://doi.org/10.1890/09-2172.1
7 Buchholz, K. & Pickering, J. L. DBH-Distribution Analysis: An Alternative to Stand-Age Analysis. *Bulletin of the Torrey Botanical Club* **105**, 282-288 (1978). https://doi.org:10.2307/2484921
8 Batjes, N. H., Ribeiro, E. & van Oostrum, A. Standardised soil profile data to support global mapping and modelling (WoSIS snapshot 2019). *Earth Syst. Sci. Data* **12**, 299-320 (2020). https://doi.org:10.5194/essd-12-299-2020
9 Hartigan, J. A. & Wong, M. A. Algorithm AS 136: A K-Means Clustering Algorithm. *Journal of the Royal Statistical Society. Series C (Applied Statistics)* **28**, 100-108 (1979). https://doi.org:10.2307/2346830
10 Hubert, L. & Arabie, P. Comparing partitions. *Journal of Classification* **2**, 193-218 (1985). https://doi.org:10.1007/BF01908075
11 Zohner, C. M. & Renner, S. S. Innately shorter vegetation periods in North American species explain native–non-native phenological asymmetries. *Nature Ecology & Evolution* **1**, 1655-1660 (2017). https://doi.org:10.1038/s41559-017-0307-3
12 Averill, C. *et al.* Alternative stable states of the forest mycobiome are maintained through positive feedbacks. *Nature Ecology & Evolution* **6**, 375-382 (2022). https://doi.org:10.1038/s41559-022-01663-9
13 Karger, D. N. *et al.* Climatologies at high resolution for the earth's land surface areas. *Scientific Data* **4**, 170122 (2017). https://doi.org:10.1038/sdata.2017.122
14 Wilson, A. M. & Jetz, W. Remotely Sensed High-Resolution Global Cloud Dynamics for Predicting Ecosystem and Biodiversity Distributions. *PLOS Biology* **14**, e1002415 (2016). https://doi.org:10.1371/journal.pbio.1002415
15 Fick, S. E. & Hijmans, R. J. WorldClim 2: new 1-km spatial resolution climate surfaces for global land areas. *International Journal of Climatology* **37**, 4302-4315 (2017). https://doi.org:10.1002/joc.5086
16 Amatulli, G. *et al.* A suite of global, cross-scale topographic variables for environmental and biodiversity modeling. *Scientific Data* **5**, 180040 (2018). https://doi.org:10.1038/sdata.2018.40
17 Venter, O. *et al.* Global terrestrial Human Footprint maps for 1993 and 2009. *Scientific Data* **3**, 160067 (2016). https://doi.org:10.1038/sdata.2016.67
18 Tuanmu, M.-N. & Jetz, W. A global 1-km consensus land-cover product for biodiversity and ecosystem modelling. *Global Ecol Biogeogr* **23**, 1031-1045 (2014). https://doi.org:https://doi.org/10.1111/geb.12182
19 Klein Goldewijk, K., Beusen, A., Doelman, J. & Stehfest, E. Anthropogenic land use estimates for the Holocene – HYDE 3.2. *Earth Syst. Sci. Data* **9**, 927-953 (2017). https://doi.org:10.5194/essd-9-927-2017
20 Fan, Y., Li, H. & Miguez-Macho, G. Global patterns of groundwater table depth. *Science* **339**, 940-943 (2013). https://doi.org:10.1126/science.1229881

21 Hengl, T. *et al.* SoilGrids250m: Global gridded soil information based on machine learning. *Plos One* **12**, e0169748 (2017). https://doi.org:10.1371/journal.pone.0169748

22 Entekhabi, D. *et al.* The Soil Moisture Active Passive (SMAP) Mission. *Proceedings of the IEEE* **98**, 704-716 (2010). https://doi.org:10.1109/JPROC.2010.2043918

23 Batjes, N. H. Harmonized soil property values for broad-scale modelling (WISE30sec) with estimates of global soil carbon stocks. *Geoderma* **269**, 61-68 (2016). https://doi.org:https://doi.org/10.1016/j.geoderma.2016.01.034

24 Klein Goldewijk, K., Beusen, A. & Janssen, P. Long-term dynamic modeling of global population and built-up area in a spatially explicit way: HYDE 3.1. *The Holocene* **20**, 565-573 (2010). https://doi.org:10.1177/0959683609356587

25 Zomer, R. J., Xu, J. & Trabucco, A. Version 3 of the Global Aridity Index and Potential Evapotranspiration Database. *Sci Data* **9**, 409 (2022). https://doi.org:10.1038/s41597-022-01493-1
