# Supplementary material for: Positive feedbacks and alternative stable states in forest leaf types
Source: Nat Commun. 2024 May 31;15:4658. doi: 10.1038/s41467-024-48676-5 (PMC11143268; doi:10.1038/s41467-024-48676-5)
Supplement: Supplementary file 2 — Reporting Summary [file 41467_2024_48676_MOESM2_ESM.pdf]

nature portfolio

Corresponding author(s): Yibiao Zou

Last updated by author(s): Apr 24, 2024

Reporting Summary

Nature Portfolio wishes to improve the reproducibility of the work that we publish. This form provides structure for consistency and transparency in reporting. For further information on Nature Portfolio policies, see our Editorial Policies and the Editorial Policy Checklist.

Statistics

For all statistical analyses, confirm that the following items are present in the figure legend, table legend, main text, or Methods section.

n/a	Confirmed	
[ ]	[x]	The exact sample size (*n*) for each experimental group/condition, given as a discrete number and unit of measurement
[x]	[ ]	A statement on whether measurements were taken from distinct samples or whether the same sample was measured repeatedly
[ ]	[x]	The statistical test(s) used AND whether they are one- or two-sided<br>*Only common tests should be described solely by name; describe more complex techniques in the Methods section.*
[ ]	[x]	A description of all covariates tested
[ ]	[x]	A description of any assumptions or corrections, such as tests of normality and adjustment for multiple comparisons
[ ]	[x]	A full description of the statistical parameters including central tendency (e.g. means) or other basic estimates (e.g. regression coefficient) AND variation (e.g. standard deviation) or associated estimates of uncertainty (e.g. confidence intervals)
[ ]	[x]	For null hypothesis testing, the test statistic (e.g. *F*, *t*, *r*) with confidence intervals, effect sizes, degrees of freedom and *P* value noted<br>*Give P values as exact values whenever suitable.*
[x]	[ ]	For Bayesian analysis, information on the choice of priors and Markov chain Monte Carlo settings
[x]	[ ]	For hierarchical and complex designs, identification of the appropriate level for tests and full reporting of outcomes
[ ]	[x]	Estimates of effect sizes (e.g. Cohen's *d*, Pearson's *r*), indicating how they were calculated

Our web collection on statistics for biologists contains articles on many of the points above.

Software and code

Policy information about availability of computer code

Data collection	No software was used.
Data analysis	The analyses were run in R [R Core Team (2021) R: a language and environment for statistical computing. R Foundation for Statistical Computing, Vienna, Austria. http://www.R-project.org. Accessed 17 Jan 2021] and Google Earth Engine (https://earthengine.google.com). The code used for this study is available at https://github.com/Yibiaozou/AltSS_ForestLeafPhenology and Zenodo (https://doi.org/10.5281/zenodo.11035706).

For manuscripts utilizing custom algorithms or software that are central to the research but not yet described in published literature, software must be made available to editors and reviewers. We strongly encourage code deposition in a community repository (e.g. GitHub). See the Nature Portfolio guidelines for submitting code & software for further information.

Data

Policy information about availability of data

All manuscripts must include a data availability statement. This statement should provide the following information, where applicable:

- Accession codes, unique identifiers, or web links for publicly available datasets
- A description of any restrictions on data availability
- For clinical datasets or third party data, please ensure that the statement adheres to our policy

The GFBi dataset will be available upon a request via Science-i (https://science-i.org/) or GFBI website (https://www.gfbinitiative.org/) and an approval from data contributors, due to data privacy laws. The FIA data of the US are publicly available from the FIA datamart (https://apps.fs.usda.gov/fia/datamart/). The

FunDivEUROPE dataset will be available upon a request via http://project.fundiveurope.eu, due to data privacy laws. All environmental covariate data sources are publicly available and detailed in the Table. S2. Source data to reproduce figures in this study are provided as a Source Data file (10.5281/zenodo.11048857).

Research involving human participants, their data, or biological material

Policy information about studies with human participants or human data. See also policy information about sex, gender (identity/presentation), and sexual orientation and race, ethnicity and racism.

Reporting on sex and gender	N/A
Reporting on race, ethnicity, or other socially relevant groupings	N/A
Population characteristics	N/A
Recruitment	N/A
Ethics oversight	N/A

Note that full information on the approval of the study protocol must also be provided in the manuscript.

Field-specific reporting

Please select the one below that is the best fit for your research. If you are not sure, read the appropriate sections before making your selection.

☐ Life sciences ☐ Behavioural & social sciences ☒ Ecological, evolutionary & environmental sciences

For a reference copy of the document with all sections, see nature.com/documents/nr-reporting-summary-flat.pdf

Ecological, evolutionary & environmental sciences study design

All studies must disclose on these points even when the disclosure is negative.

Study description	The emergence of alternative stable states in forest systems has significant implications for the functioning and structure of the terrestrial biosphere, yet empirical evidence remains scarce. Here, we combine global forest biodiversity observations (815578 GFBi plots, 45276 FIA plots and 15431 FunDivEurope plots) and simulations to test for alternative stable states in the presence of evergreen and deciduous forest types. We reveal a bimodal distribution of forest leaf types at continental (North America and Europe) and global scales that cannot be explained by environment alone, suggesting signatures of alternative forest states. Moreover, we used generalized additive model to empirically demonstrate the existence of positive feedbacks in tree growth, recruitment and mortality, with trees having 11-37% higher growth rates, 15-19% higher survival rates and 3-7 folds higher recruitment rates when they are surrounded by trees of their own leaf type. Data-driven simulations show that the observed positive feedbacks are necessary and sufficient to generate alternative forest states, which may also lead to dependency on history (hysteresis) during ecosystem transition from evergreen to deciduous forests and vice versa. We identify hotspots of bistable forest types in evergreen-deciduous ecotones and at the poleward range limits of forests, which are likely driven by soil conditions. We then map global distribution of forest bimodality using spatial random forest modelling. These findings are integral to predicting the distribution of forest biomes, and aid to our understanding of global biodiversity, carbon turnover and terrestrial climate feedbacks.
Research sample	Plot-level forest leaf phenology types across the globe. The GFBi dataset will be available upon a request via Science-i (https://science-i.org/) or GFBI website (https://www.gfbinitiative.org/) and an approval from data contributors, due to data privacy laws. The FIA data of the US are publicly available from the FIA datamart (https://apps.fs.usda.gov/fia/datamart/). The FunDivEUROPE dataset will be available upon a request via http://project.fundiveurope.eu, due to data privacy laws. All environmental covariate data sources are publicly available and detailed in the Table. S2. Source data to reproduce figures in this study are provided as a Source Data file (10.5281/zenodo.11048857).
Sampling strategy	No statistical methods were used to predetermine sample size. As we used all the data in the three dataset.
Data collection	Tree occurrence data came from the Global Forest Biodiversity initiative (GFBi) dataset, the Forest Inventory and Analysis (FIA) program of the U.S. and the FunDivEUROPE dataset. Information on leaf habit (evergreen vs. deciduous) and leaf form (broadleaved vs. needle-leaved) came from the TRY plant trait database.
Timing and spatial scale	Time: the average year of observation across all plots was 2005. The average period in between two census for FIA plots is ~5 years. The average period in between two census for FunDivEurope plots is ~10 years.<br>Spatial: global and continental scale.
Data exclusions	Individuals with stem diameters <10 cm were excluded as the focus was on adult trees, and only plots with ≥10 adult individuals were included in the final analysis.
Reproducibility	All attempts to repeat the analysis were successful.
Randomization	Not relevant. As all group allocation are based on certain classes (such as known functional traits).

Blinding	Not relevant. As data were measured on trees, which didn't involve human.

Did the study involve field work? ☐ Yes ☒ No

Reporting for specific materials, systems and methods

We require information from authors about some types of materials, experimental systems and methods used in many studies. Here, indicate whether each material, system or method listed is relevant to your study. If you are not sure if a list item applies to your research, read the appropriate section before selecting a response.

Materials & experimental systems

n/a	Involved in the study
☒	☐ Antibodies
☒	☐ Eukaryotic cell lines
☒	☐ Palaeontology and archaeology
☒	☐ Animals and other organisms
☒	☐ Clinical data
☒	☐ Dual use research of concern
☒	☐ Plants

Methods

n/a	Involved in the study
☒	☐ ChIP-seq
☒	☐ Flow cytometry
☒	☐ MRI-based neuroimaging
